# *Bph32*, a novel gene encoding an unknown SCR domain-containing protein, confers resistance against the brown planthopper in rice

**DOI:** 10.1038/srep37645

**Published:** 2016-11-23

**Authors:** Juansheng Ren, Fangyuan Gao, Xianting Wu, Xianjun Lu, Lihua Zeng, Jianqun Lv, Xiangwen Su, Hong Luo, Guangjun Ren

**Affiliations:** 1 Crop Research Institute, Sichuan Academy of Agricultural Sciences, Chengdu, 610066, P.R. China; 2 Department of Genetics and Biochemistry, Clemson University, 110 Biosystems Research Complex, Clemson, SC 29634-0318, USA; 3 Sichuan Normal University, Chengdu, 610066, P.R. China

## Abstract

An urgent need exists to identify more brown planthopper (*Nilaparvata lugens* Stål, BPH) resistance genes, which will allow the development of rice varieties with resistance to BPH to counteract the increased incidence of this pest species. Here, using bioinformatics and DNA sequencing approaches, we identified a novel BPH resistance gene, LOC_Os06g03240 (MSU LOCUS ID), from the rice variety Ptb33 in the interval between the markers RM19291 and RM8072 on the short arm of chromosome 6, where a gene for resistance to BPH was mapped by Jirapong Jairin *et al.* and renamed as “*Bph32*”. This gene encodes a unique short consensus repeat (SCR) domain protein. Sequence comparison revealed that the *Bph32* gene shares 100% sequence identity with its allele in *Oryza latifolia*. The transgenic introgression of *Bph32* into a susceptible rice variety significantly improved resistance to BPH. Expression analysis revealed that *Bph32* was highly expressed in the leaf sheaths, where BPH primarily settles and feeds, at 2 and 24 h after BPH infestation, suggesting that *Bph32* may inhibit feeding in BPH. Western blotting revealed the presence of Pph (Ptb33) and Tph (TN1) proteins using a Penta-His antibody, and both proteins were insoluble. This study provides information regarding a valuable gene for rice defence against insect pests.

Rice (*Oryza sativa* L.) is the primary source of calories for more than one-third of the world’s population, particularly in Asia. Rice crops are often attacked by many diseases and insects, resulting in substantial yield losses. Since the 1960 s, among insect pests, the brown planthopper (*Nilaparvata lugens* Stål, BPH) has become one of the most important pests in the rice-growing areas of Asia^1^. BPH not only directly damages rice by sucking phloem sap and by ovipositing in plant tissues, but also transmits viral diseases such as grassy stunt virus (RGSV) and ragged stunt virus (RRSV)^2^. Although different trends of damage have been observed in Asian countries, problems involving rice planthopper are intensifying^3^,^4^. The excessive use of insecticides has led to the development of insecticide resistance in BPH and has disrupted the ecological balance in the rice ecosystem, representing a key factor in the increased incidence of the BPH^3^,^5^,^6^.

A sound balance between breeding for resistance and pest management for BPH management is important; the objective of such management is to reduce the ecological fitness of the BPH, thereby keeping its numbers below economic threshold levels^7^. However, frequent changes in the biotypes and populations of BPH are among the major challenges for rice breeders. For example, the BPH resistance genes *Bph1* and *bph2* rapidly became obsolete in just 3–5 years because of the development of new BPH biotypes^7^. Since the first reports of the BPH resistance genes *Bph1* and *bph2* by Athwal *et al.*^8^, at least 30 new BPH resistance loci have been identified from cultivated and wild species of *Oryza*^7^,^9^,^10^,^11^,^12^,^13^,^14^,^15^,^16^,^17^,^18^. To date, four of these resistance genes, *Bph14*^19^, *BPH26*^11^, *Bph3*^20^ and *BPH29*^12^, have been cloned. *Bph14* and *BPH26* both encode a coiled-coil, nucleotide-binding-site, leucine-rich repeat (CC-NBS-LRR) protein^19^,^21^. *BPH29* contains a B3 DNA-binding domain^12^. Three BPH resistance genes were inferred to activate the salicylic acid signaling pathway^11^,^12^,^19^. *Bph3* is a cluster of three BPH resistance genes encoding plasma membrane-localized lectin receptor kinases (OsLecPK1- OsLecPK3)^20^. These studies provide insights into the molecular mechanisms of plant-insect interactions and serve as resources for molecular BPH-resistant rice breeding.

The gene *Bph3*, was first designated by Lakshminarayana and Khush and identified in the rice variety Rathu Heenati^22^. Subsequent studies have found that *Bph3* displays resistance to four BPH biotypes^23^,^24^ and is currently still effective despite more than 30 years of deployment^20^,^25^,^26^,^27^. However, the map position of the *Bph3* locus on the rice chromosome has been disputed. The *Bph3* gene has primarily been reported to be either allelic or tightly linked to another BPH resistance gene, *bph4*, in the rice variety Babawee^28^. This allelic relation was further confirmed by Angeles *et al.*^29^, and these two allelic BPH resistance genes were first located on chromosome 7 according to trisomic analysis^30^. However, the *Bph3* gene was subsequently found to be physically located on chromosome 4 based on florescence *in situ* hybridization^31^. Although a major BPH resistance gene, tentatively referred to as *Bph17*, was identified from the rice variety Rathu Heenati on chromosome 4^32^, the gene that was thereafter named *Bph3*, was cloned by Yuqiang Liu *et al.*^20^. However, Jirapong Jairin *et al.* mapped the *Bph3* gene to the short arm of chromosome 6 using two backcross populations, BC_1_F_2_ and BC_3_F_2_, from crosses of Ptb33 × RD6 and Rathu Heenati × KDML105^33^. Furthermore *Bph3* was physically mapped to an approximately 190-kb interval flanked by the markers RM19291 and RM8072^34^. The BPH resistance locus has widely been used in BPH resistance breeding via marker-assisted selection (MAS)^35^,^36^,^37^, revealing that the locus contains another valuable BPH resistance gene.

In the present study, a dominant gene against BPH, *Bph32*, was cloned from the rice variety Ptb33 in an approximately 190-kb interval flanked by the markers RM19291 and RM8072 on the short arm of chromosome 6 using bioinformatics analysis and a transgenic approach. The evaluation of BPH resistance in transgenic plants confirmed the crucial function of *Bph32* in BPH resistance. *Bph32* encodes a short consensus repeat (SCR) domain-containing protein that confers an antibiosis resistance to BPH and is localized in the plasma membrane of the cell. This gene is highly expressed in the leaf sheaths, where the BPH first settles and feeds. The results confirm that *Bph32* is a stable BPH resistance gene and provides a valuable gene for rice defence against insect pests.

## Results

### Identification of *Bph32*

**Figure 1:**
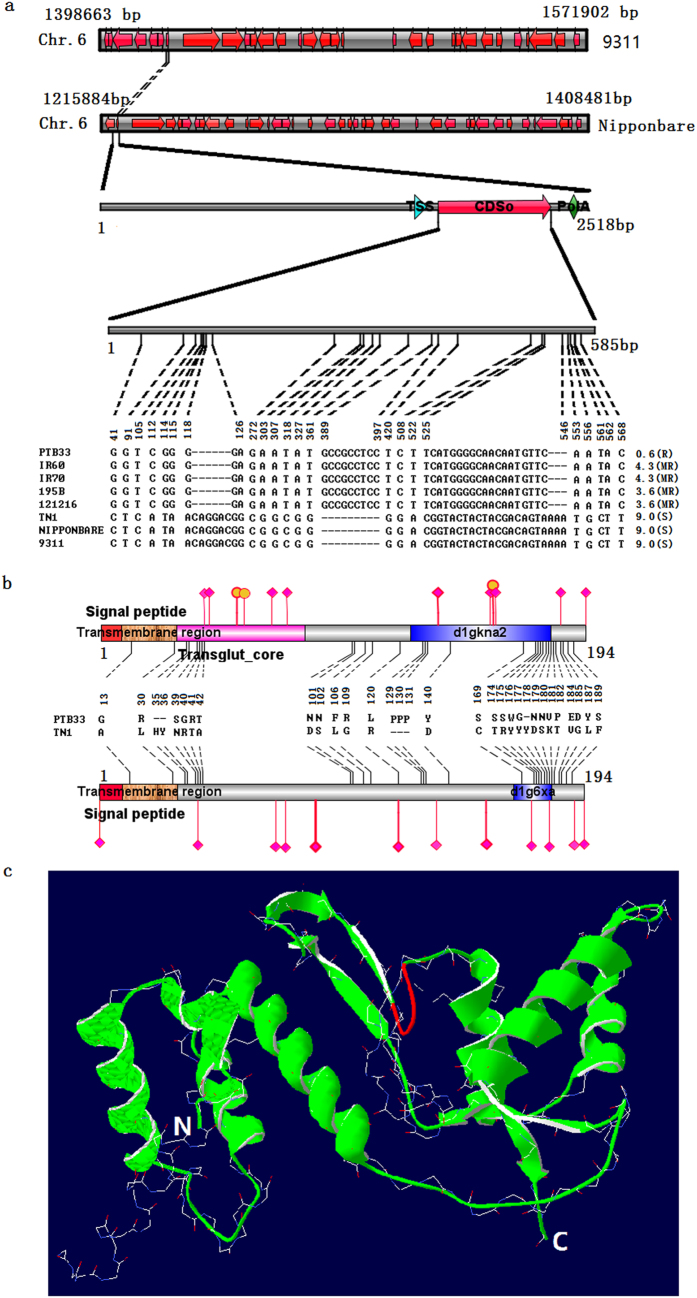
Identification of *Bph32* using bioinformatics and DNA sequencing. (**a**) Comparison of the genomic fragments flanked by the SSR markers, RM19291 and RM8072 in the 9311 and Nipponbare genomes, and comparison of the cDNA sequences of the LOC_Os06g03240 gene in Ptb33, IR60, IR70, 195B, 121216, TN1, Nipponbare and 9311 revealed that all of these resistant varieties/lines have identical nucleotides and several nucleotide polymorphisms and multiple nonsynonymous nucleotide polymorphisms in the susceptible TN1, Nipponbare and 9311 lines. (**b**) Comparison of the LOC_Os06g03240 protein sequences in Ptb33 and TN1 revealed the presence of amino acid substitutions, deletions, insertions and different protein domains and binding sites between the two varieties. (**c**) The 3D protein structure of LOC_Os06g03240 in Ptb33 based on a template, 3DEE_A, with a −9.19 Z-Score using Alignment Mode. Six α-helixes and six β-sheets are present.

To identify the *Bph32* gene, an approximately 190-kb nucleotide sequence from Nipponbare and an approximately 170-kb nucleotide sequence from 9311, flanked by the simple sequence repeat (SSR) markers RM19291 and RM8072, were analysed using bioinformatics methods. Totals of 39 and 31 putative genes were identified from the 190 and 170-kb fragments, respectively, using the Fgenesh gene-finder^38^ at http://linux1.softberry.com/berry.phtml?topic=fgenesh&group=programs&subgroup=gfind (Table S1). Eight previously reported putative genes^34^ were removed from the predicted putative genes. The protein domains of 31 remaining genes were detected using SMART^39^,^40^ at http://smart.embl-heidelberg.de/ (Table S1). The LOC_Os06g03240 (MSU LOCUS ID) encoding 194 amino acids was chosen because its protein contained a scop dig6xa_ domain, which was described as a Kunitz/Bovine pancreatic trypsin inhibitor domain (Table S2a and Fig. 1a).

Sequence comparison of the LOC_Os06g03240 exons was performed for the resistant varieties Ptb33, IR60, IR70, 195B, and 121216 and for the susceptible varieties, Taichung Native (TN1), Nipponbare and 9311. The LOC_Os06g03240 gene had an identical sequence in all the resistant varieties/lines but displayed several nucleotide polymorphisms compared with the susceptible TN1, Nipponbare and 9311. These polymorphisms included, two deletions of 6 bp(119–124 bp) and 3 bp(544–546 bp); a 9-bp insertion between 389 and 397 bp; and multiple nonsynonymous nucleotide polymorphisms, such as G/C in the 41^st^ bp, G/T in the 91^st^ bp, and T/G in the 105^th^ bp (Fig. 1a and Figure S1). Moreover, short insertions and deletions of several nucleotides (InDels) were detected in the promoter of LOC_Os06g03240 between Ptb33 and TN1 (Figure S1). Sequence comparison of the LOC_Os06g03240 protein between Ptb33 and TN1 revealed 25 amino acid substitutions, 2 deletions and one 3-amino-acid insertion (Fig. 1b and Figure S2). The LOC_Os06g03240 gene in Ptb33 encodes an unknown protein with a molecular weight (MW) of 21658.34 Da and an isoelectric point (pI) of 7.71. This protein contains a signal peptide and a SCOP (Structural Classification of Proteins) d1gkna2 domain belonging to the complement control module/SCR domain, which is considered to be a type of lectin or cell adhesion protein^41^. Plant lectin are among the most important direct defence proteins in plants against attack by insect pests^42^. The LOC_Os06g03240 protein of Ptb33 contains seven protein-binding (SO:0000410)^43^,^44^ and three polynucleotide-binding regions (SO:0001429)^44^,^45^ (Table S2b and Fig. 1b). The LOC_Os06g03240 gene in TN1 encodes a protein with an MW of 21,789.62 Da and a pI of 7.67, that contains twelve protein-binding regions (SO:0000410)^43^,^44^ (Table S2b and Fig. 1b). The two LOC_Os06g03240 proteins from Ptb33 and TN1 are predicted to be localized to the plasma membrane of the cell^46^.

Based on a template, 3DEE_A, the 3D structure of the LOC_Os06g03240 protein in Ptb33 was successfully modeled with a Z-Score of −9.19^47^ using the Alignment Mode of the SWISS-MODEL workspace. This 3D structure contains six α-helixes and six β-sheets (Fig. 1c). However, an attempt to model the 3D structure of the LOC_Os06g03240 protein in TN1 using the Alignment Mode was unsuccessful. These observations led us to speculate that the LOC_Os06g03240 gene, renamed as “*Bph32*” in this study, might be a candidate gene for conferring BPH resistance.

### *Bph32* confers an antibiosis resistance to BPH

**Figure 2:**
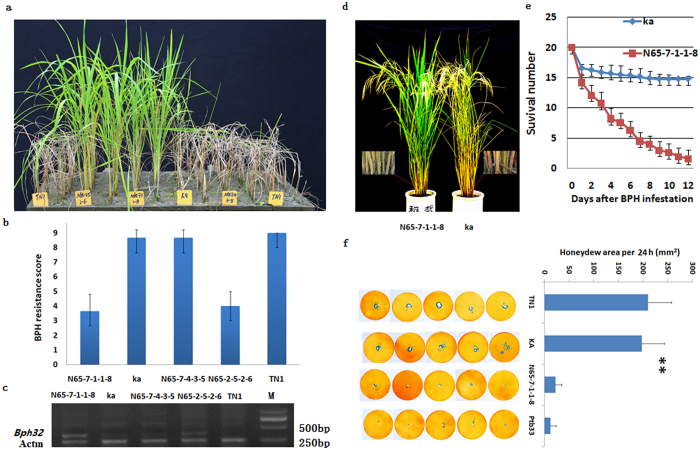
Complementation test of the *Bph32* gene and characterization of BPH resistance in *Bph32* transgenic rice. (**a**) BPH resistance test of *Bph32* transgenic and susceptible wild-type (WT) rice. TN1, susceptible variety (CK); Ka, susceptible WT rice; N65-7-4-3-5, empty-vector transgenic T_2:3_ line; N65-2-5-2-6 and N65-7-1-1-8, *Bph32* transgenic T_2:3_ lines. (**b**) BPH resistance scores of *Bph32* transgenic T_2:3_ lines using the modified standard seedbox screening. The data are presented as means ± SD (three replications). (**c**) RT-PCR analysis showing *Bph32* expression in the transgenic T_2:3_ lines. (**d**) BPH resistance test of the *Bph32* transgenic plants (N65-7-1-1-8) and susceptible wild-type (WT) plants (Ka) at the mature stage. Magnified views showed the locations of BPH feeding. (**e**) BPH survival number in the *Bph32* transgenic plants (N65-7-1-1-8) and susceptible wild-type (WT) plants (Ka) from the first to the twelfth days after BPH infestation. (**f**) Comparison of the honeydew area in TN1, Ka, N65-7-1-1-8 and Ptb33 using the honeydew excretion test. **P < 0.01. One-way ANOVA was used to generate the P value.

To confirm that LOC_Os06g03240 confers BPH resistance, we transformed a susceptible *indica* variety, Kasalath (Ka), with the cDNA sequence of the Ptb33 *Bph32* gene. Six independent transgenic events were detected using genomic southern blotting. Among them, N65-7-1-1-8 contains a single-copy of *Bph32* and N65-2-5-2-6 contains a double-copy. Their T_2:3_ plants were used to measure the levels of resistance of *Bph32*. As demonstrated in Fig. 2, upon infestation with BPH at the seedling stage, all the wild-type control (Kasalath and TN1) and empty-vector transgenic plants died, whereas the transgenic rice plants expressing the Ptb33 *Bph32* gene survived (Fig. 2a,b and c). When infected at the maturing stage, the wild-type plants exhibited leaf wilting, a decrease in seed and grain plumpness, and even death of the whole plant, whereas the *Bph32* transgenic plants were all healthy (Fig. 2d).

Plants have evolved three defence mechanisms for responding to insect attack: antixenosis repels or disturbs insect settling, thereby reducing colonization or oviposition; antibiosis reduces insect feeding, survival or growth rate; and tolerance helps a crop to maintain high quality and yield after insect infestation^48^. To explore how *Bph32* is involved in plant resistance to BPH, we conducted assays to compare nymph survival and honeydew excretion between the resistant *Bph32* transgenic and susceptible wild-type plants (Table S3 and Fig. 2e and f). In the nymph survival test, the number of nymphs steadily decreased on the transgenic plants but began to stabilize 8 days after infestation on the wild-type controls (Fig. 2e). Honeydew excretion in the honeydew area of the transgenic plants was significantly lower than that of the wild-type controls or TN1 plants. However, the difference in honeydew excretion between the transgenic plants and the Ptb33 plants was insignificant (Fig. 2f). These results demonstrate that BPH feeding was inhibited on the resistant *Bph32* transgenic plants and suggest that *Bph32* conferred an antibiosis resistance that reduced BPH feeding and survival.

### Expression analysis of *Bph32* and subcellular localization of the Bph32 protein

**Figure 3:**
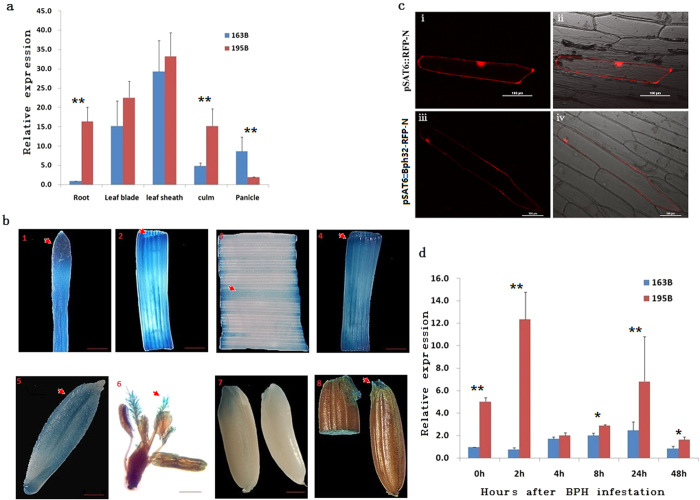
*Bph32* expression analysis. (**a**) Comparison of *Bph32* expression as measured by RT-PCR in different organs in BPH-resistant 195B (red bar) and BPH-susceptible163B (blue bar). (**b**) *Bph32* promoter-*GUS* expression pattern in transgenic rice plants. GUS expression profiles in root, culm, leaf blade, leaf sheath, glume, flower, immature seed and germinating seed, respectively (scale bars are as follows: 1–4, 500 μm; 5, 7 and 8, 800 μm; 6, 100 μm). (**c**) *Bph32* subcellular localization. i–ii, Localization of the empty vector. Fluorescence (i) and merged image (ii) of the red fluorescence channel is shown in the top panel. iii–iv, Onion epithelial cells expressing the Bph-ptb33 fusion protein. Fluorescence (iii) and merged (iv) images showing that the Bph32 protein was localized mainly in the plasma membrane. (Scale bar: 100 μm) (**d**) Comparison of *Bph32* expression by RT-PCR after BPH infestation in 195B (red bar) and 163B (blue bar). **P < 0.01; *P < 0.05. One-way ANOVA was used to generate the P value.

To reveal the molecular mechanisms underlying *Bph32*-mediated BPH resistance, we examined the expression profile of the *Bph32* gene. Real-time (RT) PCR analysis showed that *Bph32* was expressed in all investigated tissues at the flowering stage, and its expression level was highest in leaf sheaths followed by leaf blades, culms, panicles and roots (Fig. 3a), consistent with the preference of BPH to settle and probe in leaf sheaths at the flowering stage^49^. *Bph32* expression was further analysed in more detail using transgenic plants carrying a *Bph32* promoter-driven *GUS* reporter gene. GUS expression was observed in the root, leaf blade, leaf sheath, culm, glume, flower, immature seed and germinating seed (Fig. 3b), and GUS activity was strongly detected in parenchyma cells and the vascular bundle (Fig. 3b1 and 3b2). Notably, the expression levels of the *Bph32* gene in some tissues of the susceptible line were different from that of the resistant line, which may be associated with the different in 5′ regulatory sequences (Figure S1a).

To confirm the subcellular localization of Bph32 protein, the *Bph32* cDNA fused to the red fluorescent protein (RFP) gene at the N-terminal end and under the control of the *CaMV 35S* promoter was transiently expressed in onion epidermal cells. Consistent with our prediction, the Bph32 protein was observed in the plasma membrane of the cells (Fig. 3c).

To investigate how *Bph32* responds to BPH infestation, we examined *Bph32* expression at 0, 2, 4, 8, 24 and 48 h after infestation using RT-PCR. *Bph32* expression in the resistant plant 195B was significantly induced compared with that in the susceptible plant 163B at 2 and 24 h after infestation (Fig. 3d). These results support the notion that the sequence variations in the coding and the promoter regions of the *Bph32* genes between the resistant and susceptible varieties accounted for the difference in gene function regarding BPH resistance (Figure S1a).

**Figure 4:**
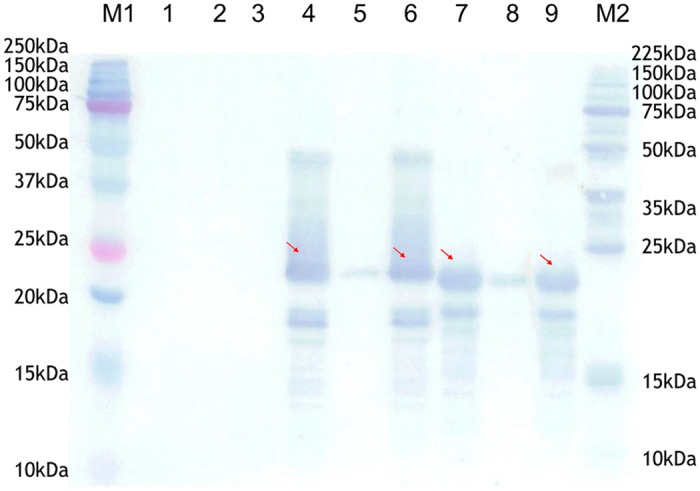
Western blotting of Bph32 protein expression in *E. coli* BL21T1R. M1, Precision Plus protein standard; 1, 2, and 3, whole cell lysate, supernatants and sediments of *E. coli* cells harbouring the control pClod l, respectively; 4, 5, and 6, whole cell lysate, supernatants and sediments of *E. coli* cells harbouring CDG0933-2(Ptb33, Pph), respectively; 7, 8, and 9, whole cell lysate, supernatants and sediments of *E. coli* cells harbouring CDG0934-1(TN1:Tph), respectively; M2, Perfect Protein Marker.

Western blotting revealed that the Pph (Pyb33) and Tph (TN1) proteins were successfully expressed in *Escherichia coli (E. coli*) BL21T1R and could be detected using a Penta-His antibody (Fig. 4). The two proteins were insoluble and differed slightly in size.

### Phylogenetic relationship among the Bph32 proteins and the characterization of 195B, a Ptb33 introgression line with BPH resistance

**Figure 5:**
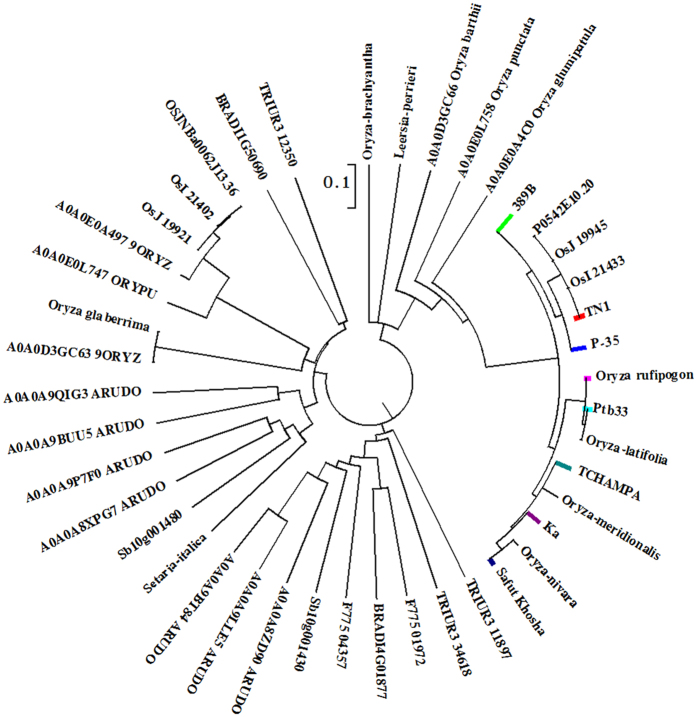
Phylogenetic relationships of *Bph32* homologs. Among 123 rice varieties and accessions, *Bph32* has eight alleles: the TN1 genotype: *bph32* (64), the Ptb33 genotype: *Bph32* (21), the 389B genotype: *bph32*^*389B*^ (15), the Kasalash genotype: *bph32*^*ka*^ (8), the *Oryza rufipogon* genotype: *bph32*^*Or*^ (7), the TCHAMPA genotype: *bph32*^*TC*^ (4), the P-35 genotype: *bph32*^*p*^ (2) and the Safut Khosha genotype: *bph32*^*SK*^ (2). The numbers in brackets represent the numbers of rice varieties and accessions with 100% identity in the *Bph32* sequence. Or, *Oryza*; Sb, *sorghum bicolor*; ARUDO, *Arundo donax*; F775, *Aegilops tauschii*; TRIUA, *Triticum urartu*; BRADI, *Brachypodium distachyon* (scale bar: 0.1 amino acid substitutions per site).

Comparison of the *Bph32* cDNA sequence of 123 rice varieties and accessions revealed the following 8 alleles of the *Bph32* sequence (Table S4): the TN1 genotype: *bph32* (64), the Ptb33 genotype: *Bph32* (21), the 389B genotype: *bph32*^*389B*^ (15), the Kasalash genotype: *bph32*^*ka*^ (8), the *Oryza rufipogon* genotype: *bph32*^*Or*^ (7), the TCHAMPA genotype: *bph32*^*TC*^ (4), the P-35 genotype: *bph32*^*p*^ (2) and the Safut Khosha genotype: *bph32*^*SK*^ (2) (the numbers in brackets are the numbers of rice varieties and accessions with 100% identity in the *Bph32* sequence). *Bph32* in Ptb33 shares 100% sequence identity with its allele in *Oryza latifolia* (Table S4, Fig. 5, Figure S3), the wild rice species in South and Central America and an important contributor to BPH resistance^17^,^50^,^51^. The levels of BPH resistance of 119 rice varieties and accessions were investigated in our screen experiment, and BPH resistance scales of the *Bph32*-containing varieties and accessions were all below 5 (Table S4). BLAST searches performed on http://www.uniprot.org/blast/ showed that 34 *Bph32* homologs are found in *Oryza* (Or), its outgroup (*Leersia perrieri*), *Sorghum bicolor* (Sb), *Arundo donax* (ARUDO), *Aegilops tauschii* (F775), *Triticum urartu* (TRIUA), *Brachypodium distachyon* (BRADI) and *Setaria italica*. Phylogenetic analysis revealed that *Bph32* encodes a unique complement control module, or SCR domain-containing protein, and is closely related to other *Oryza* homologs and other Gramineae crops (Fig. 5).

**Figure 6:**
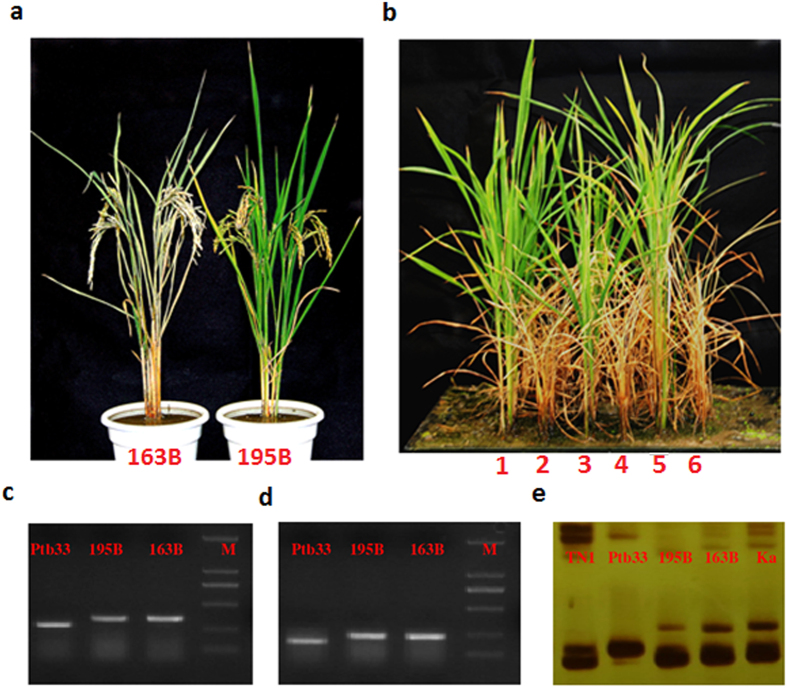
BPH resistance of the maintainer line 195B and its combinations by genotypic analysis and phenotypic reaction. (**a,b**) BPH resistance test of 195B at the mature and seedling stages. 1, 2, 3, 4, 5 and 6 represent 195 A/Chenghui3203, Chenghui3203, 195B, 163 A/Chenghui3203, Ptb33 and TN1, respectively. (**c–e**) Gene marker analysis of 195B. (**c,d**), Gene markers Bph3-In1 and Bph3-In2 for *Bph3*; (**e**) gene marker Bp26-INd for *BPH26*.

**Figure 7:**
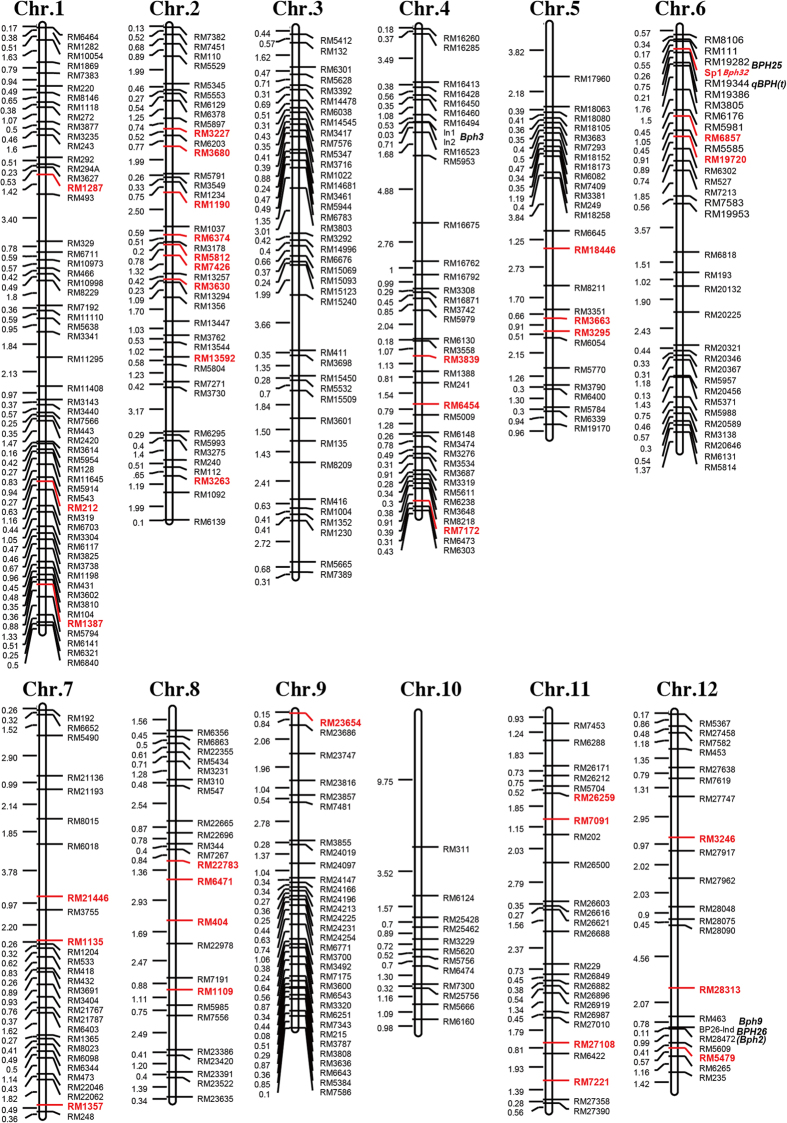
Genotypic analysis of the 195B line. The 195B line contained 36 Ptb33 polymorphic SSR loci (red font) or 21.95% Ptb33 genetic background, and one BPH resistance gene *Bph32* (the 115 F2:3 lines of a 195B and 106B (susceptible) cross showed a fit to 1:2:1 (26:60:29) ratio (χ^2^ = 0.19, P = 0.91) for resistant, segregating and susceptible). *Bph3*^20^, *BPH25*^10^, *qBPH(t*)^57^, *BPH26(Bph2*)^11^, and *Bph9*^82^,^83^ are marked on this genetic map based on previous reports.

To utilize *Bph32* in BPH resistance breeding, the maintainer line 195B (F_14_) containing *Bph32* was selected from the progeny of a backcross Ptb33/163B//163B through marker-assisted selection and insect identification. The BPH resistance of 195B and its combination was stable, and the average scores of BPH resistance were both 3.6 (Tables S3 and S4, Fig. 1a, Fig. 6a and b). The genetic background of the line 195B was analysed using 426 SSR markers and BPH resistance gene markers. Among the 426 SSR markers, 371 produced DNA fragments, but only164 SSR markers (44.2% of the markers) amplified polymorphic fragments in Ptb33 and 163B. The line 195B contained 36 Ptb33 polymorphic SSR loci or 21.95% with the Ptb33 genetic background (Table S5 and Fig. 7). In previous reports, Ptb33 has been reported to have two or three BPH resistance genes (*bph2* and *Bph3* or *bph2*, *Bph3* and *Bph9*)^52^,^53^,^54^,^55^. Using gene markers and gene resequencing, we confirmed that Ptb33 contains the three genes *Bph32*, *Bph3*^*p*^ (an allele of *Bph3*) and *BPH26 (bph2*), which are associated with BPH resistance (data to be reported elsewhere), whereas 195B contained only one gene, *Bph32* (Fig. 6c,d and e, Fig. 7, Figure S4). The 115 F_2:3_ lines of a population derived from a cross between 195B and 106B(susceptible), showed a fit to a 1:2:1 (26:60:29) ratio (χ^2^ = 0.19, P = 0.91) for resistant, segregating and susceptible, further confirming that BPH resistance is under the control of a single gene. These results demonstrate that *Bph32* is a stable and dominant BPH resistance gene and is functional in conferring BPH resistance (Table S3 and S4, Fig. 6a and b).

## Discussion

### *Bph32*, a novel BPH resistance gene, encodes an unknown SCR domain-containing protein

To date, some BPH resistance genes and a BPH quantitative resistance locus (QRL) have been found on the short arm of rice chromosome 6^16^: *Bph3*^33^,^34^, *bph4*^56^, *BPH25*^10^ (*bph20(t*)^15^) and *qBPH(t*)^57^. The rice variety Ptb33 showed a higher degree and a broader spectrum of BPH resistance than has been described previously^24^,^29^,^33^,^58^. E.R. Angeles *et al.* first confirmed that Ptb33 contains two BPH resistance genes: *Bph3* and *bph2*^29^. The *bph2* gene was mapped within a 1.0-cM region delimited by two AFLP markers, KAM3 and KAM5, and was identified as a single dominant gene^59^. Later research confirmed that the sequence of *bph2* derived from ASD7 is completely identical to the sequence of *BPH26* derived from ADR52^11^. We also confirmed that the sequence of *bph2* derived from Ptb33 was completely identical to the sequence of *BPH26*^11^ using gene markers (Fig. 6e) and gene resequencing (data not shown). An allele of *Bph3*^20^ was also identified in Ptb33 using the same approach (Fig. 6c and d) (data to be reported elsewhere). The quantitative resistance locus (QRL) *qBPH(t*), unlike Ptb33, containing the BPH resistance locus on chromosome 6, was identified in IR71033-121-15, and is flanked by the SSR markers RM469 and RM586^57^. The *bph20(t*) gene was renamed *BPH25* by Myint *et al.*^10^ Although *BPH25* and *Bph32* are located in similar positions, they did not seem to be allelic because we did not find alleles against BPH (Figure S3). The BPH resistance gene flanked by the markers RM19291 and RM8072 was renamed *Bph32* because *Bph3* has already been designated.

Our results reveal that the BPH resistance gene *Bph32* is a novel gene that encodes an unknown protein containing a signal peptide and a SCOP d1gkna2 domain (Table S2a and Fig. 1b). The SCOP d1gkna2 domain belongs to the complement control module/SCR domain family, a family of cell adhesion molecules (CAMs) that are considered to be types of lectin, or cell adhesion proteins^41^. Plant lectins have previously been reported to function as direct defence proteins that can act on insect glycoproteins or tissues to inhibit insect feeding^42^,^60^. In the present study, honeydew excretion was much lower in *Bph32* transgenic plants than wild-type control plants (Fig. 2f). The 3D protein structure of *Bph32* was successfully modeled based on a template (3DEE_A) from Neisseria gonorrhoeae Fa 1090^61^ (Fig. 1c). The protein was predicted, and subsequently confirmed, by a subcellular localization experiment, to be localized to the plasma membrane (Fig. 3c). Expression analysis showed that Bph32 was highly expressed in the leaf sheaths, where BPH primarily settles and feeds^49^ (Fig. 3a). Bph32 was also highly expressed at 2 h and 24 h after BPH infestation (Fig. 3d), which suggested that *Bph32* might inhibit BPH feeding (Fig. 2f). The interaction between rice and the BPH mirrors the co-evolution between plants and their natural enemies^62^,^63^. Unlike *Bph14*, *BPH26*, *Bph3* and *BPH29*, *Bph32* encodes an unkown SCR domain-containing protein, thus providing a new insight into the molecular mechanisms underlying plant defences against insect pests.

The Pph (Pyb33) and Tph (TN1) proteins were detected using a Penta-His antibody. The observed difference in migration between the two proteins might be due to differences in their insolubility (Fig. 4), and the two proteins are currently being purified for further analysis of their bioactivity.

### *Bph32*, a stable and dominant BPH resistance gene, offers a resource for rice BPH resistance breeding

Since 1982, several Ptb33-derived, BPH-resistant rice varieties including IR60, IR62, and IR70, have been released, most of which presumably contain the *Bph3* locus for resistance based on results from laboratory BPH biotypes in seedbox tests^25^. The *Bph3* locus was successively mapped to rice chromosomes 7^30^, 4^31^ and 6^33^,^34^ and was eventually cloned from rice chromosome 4^20^. However, the BPH resistance locus, on the short arm of Ptb33 chromosome 6, was confirmed through SSR markers^35^,^36^,^37^ and was verified in the present study to be the *Bph32* gene, which is stable and dominant for BPH resistance (Tables S3 and S4, Figs 1a, 2 and Fig. 6a and b).

The *Bph32* gene shares 100% sequence identity with its allele in *Oryza latifolia* (Figure S3). *Oryza latifolia*, with a CCDD genome, is distributed throughout South and Central America and is an important contributor to BPH resistance^17^,^50^,^51^. Phylogenetic analysis of *Bph32* showed that high levels of natural variation exist in *Oryza* (Fig. 5). However, only the Ptb33 genotype controls BPH resistance. Host genetic background influences the function of the resistance genes^64^. Different levels of BPH resistance were found for different materials (Table S4). Because of the frequent change in BPH biotypes and the occurrence of insecticide resistance in BPH, rice planthopper problems are intensifying. Thus, we need to develop rice varieties that exhibit stable and durable resistance to BPH by pyramiding multiple resistance genes using MAS. Characterization of the BPH resistance gene *Bph32* should greatly assist efforts to develop and deploy rice varieties that exhibit stable and durable resistance to BPH.

## Methods

### Plant and insect materials

The 123 varieties of cultivated rice and rice accessions used in this study are listed in Table S4 together with their names, countries of origin, taxa and the levels of resistance to brown planthopper (BPH). Of these 123 varieties, the rice variety Ptb33 shows a higher degree and a broader spectrum of BPH resistance than has been described previously^58^, and the rice variety Ptb33 is the donor of a novel BPH resistance gene, *Bph32*. The maintainer line 195B containing *Bph32* is an F_14_ selection from the progeny of the backcross Ptb33/163B//163B, and was developed using marker-assisted selection and insect identification. In this cross, the line 163B was found susceptible to BPH. The mixed biotype BPH populations used for infestation were collected from a rice field in Hainan Province, China, and were reared on plants of the susceptible variety Taichung Native (TN1) in a greenhouse maintained under a light regime of 15/9-h light/dark and day/night temperatures of 26–32 °C^33^.

### Identification of *Bph32* using bioinformatics and DNA sequencing

Ptb33, an local Indian variety carrying multiple BPH resistance genes, shows a broad-spectrum resistance against all BPH populations and has often been used to verify resistance^25^,^26^,^27^,^33^. Ptb33 carries the dominant BPH resistance gene *Bph3*^22^,^65^. Jirapong Jairin *et al.* mapped the *Bph3* locus between two flanking SSR markers, RM589 and RM588, on the short arm of chromosome 6^33^. Subsequently, the researchers localized the *Bph3* gene to a 190-kb interval flanked by the SSR markers RM19291 and RM8072^34^. An approximately 190-kb nucleotide sequence of Nipponbare flanked by the SSR markers RM19291 and RM8072 was downloaded from the GenBank database (http://rice.plantbiology.msu.edu/cgi-bin/gbrowse/rice/). Another approximately 170-kb nucleotide sequence of the *Oryza sativa* Indica Group was obtained from the Gramene database (http://archive.gramene.org/Oryza_indica/Info/Index). Totals of 39 and 31 putative genes were identified from the two indicated nucleotide sequence fragments, respectively, using the Fgenesh gene-finder^38^ at http://linux1.softberry.com/berry.phtml?topic=fgenesh&group=programs&subgroup=gfind. Eight previously reported putative genes^34^ were removed from the predicted putative genes. The protein domains of the remaining genes were detected using SMART^39^,^40^ at http://smart.embl-heidelberg.de/. We found that LOC_Os06g03240 (MSU LOCUS ID) contained a SCOP dig6xa_ domain, which was described as a Kunitz/Bovine pancreatic trypsin inhibitor domain. The genomic sequences of LOC_Os06g03240 were further analysed using DNA sequencing in Ptb33, IR70 and IR60 (two *Indica* varieties known to harbour *Bph3*)^7^ and two newly identified rice lines, 195B and 121216, whose BPH resistance is derived from Ptb33, along with the susceptible variety TN1. The genomic DNA fragment (including the promoter regions, the entire CDS region and the downstream sequence) of LOC_Os06g03240 (~2.5 kb) was amplified from Ptb33 seedling DNA using specific primers (Table S6), and the PCR product was verified by DNA sequencing. The predicted protein sequence characteristics of the product, such as MW, pI, and binding sites, were calculated using ExPASy^66^ (http://web.expasy.org/cgi-bin/compute_pi/pi_tool) and the PredictProtein server^44^ (https://www.predictprotein.org/home). To model the 3D structure of LOC_Os06g03240, a BLAST search was first performed to identify suitable templates in the Protein Data Bank (pdb) (http://blast.ncbi.nlm.nih.gov/Blast.cgi); thereafter the 3D protein structure was modelled on the SWISS-MODEL workspace using the Alignment Mode^67^,^68^,^69^ (http://www.swissmodel.expasy.org/), and was visualized using the program DeepView^70^. LOC_Os06g03240 was ultimately considered a candidate gene and was named “*Bph32*” in this study because the *Bph3* on chromosome 4 has previously been cloned by Yuqiang Liu *et al.*^20^, and the latest new *BPH* gene has been designated as *Bph31* by G.D. Prahalada *et al.*^71^.

### Plasmid construction and Western blotting

Total RNA was extracted from Ptb33 seedlings using the TaKaRa MiniBEST plant RNA Extraction Kit (TaKaRa Biotechnology (Dalian) Co., Ltd) according to the manufacturer’s instructions. To construct plasmids for the complementation test, the entire CDS fragment of *Bph32* (585 bp) was amplified from Ptb33 seedling total RNA using gene-specific primers (Table S6) from Ptb33 seedling total RNA, and the PCR product was inserted into the binary vector PHB (provided by Shanghai Jiao Tong University) to generate PHB-*Bph32*.

For subcellular localization, the *Bph32* CDS sequence was cloned into the vector pSAT6::RFP-N (purchased from https://www.arabidopsis.org/) and fused with the N-terminus of RFP, resulting in the Bph32-RFP fusion protein-expressing plasmid pSAT6::Bph32-RFP-N. Then, onion epidermal cells were transformed with the OsBph32-RFP gene via particle bombardment. Confocal images were taken 16 h after bombardment using a Nikon A1R-si laser scanning confocal microscope. The experiment was repeated three times.

To prepare the β-glucuronidase (*GUS*) reporter gene construct, a 786-bp genomic fragment, *PtP*, corresponding to the 5′ sequence upstream of the ATG start site in the *Bph32* gene was amplified from Ptb33 genomic DNA using specific primers, and the PCR product was cloned into the P1300 + PB1101 vector (provided by Shanghai Jiao Tong University) to produce transcriptional fusion, P1300 + PB1101 + *PtP*, with the *GUS* gene. All constructs were verified by DNA sequencing.

To investigate the prokaryotic expression of the *Bph32* gene, two 594-bp synthetic DNA sequences, Pph (Ptb33:*Bph32*) and Tph (TN1:*Bph32*; including the entire CDS region of *Bph32*), synthesized by TaKaRa Biotechnology (Dalian) Co., Ltd, were each subcloned into the prokaryotic expression vector pColdI and confirmed by DNA sequencing. The corresponding recombinant expression vectors were named CDG0933-2 (Pph) and CDG0934-1 (Tph). Then CDG0933-2, CDG0934-1 and control pColdI were introduced into the bacterial host *Escherichia coli (E. coli*) BL21T1R following standard protocols, and expression of the fusion genes was induced with 1 mM isopropyl-β-D-thiogalactopyranoside (IPTG) at 15 °C for 22 h or until the OD600 was approximately equal to two. *E. coli* cells that harboured the target plasmid were suspended in 320 μl of PBS in an appropriate final volume, ultrasonically crushed, and separated by centrifugation at 12,000 rpm for 10 min. Finally, three extract fractions (whole *E. coli* cell lysate, supernatant and sediment) were lysed in SDS sample buffer for 10 min at 99 °C. The lysates were analysed using 15% sodium dodecyl sulphate polyacrylamide gel electrophoresis (SDS-PAGE); the proteins were subsequently stained with Coomassie brilliant blue (CBB) R-250 for visualization.

To detect the Bph32 protein, Western blotting was performed as follows. The lysates were subjected to SDS-PAGE, after which the proteins were transferred to a polyvinylidene difluoride (PVDF) membrane. The membrane was incubated first overnight at 4 °C in 10 ml blocking buffer containing 1.5% bovine serum albumin (BSA) and then in 5 ml Penta-His antibody solution containing the primary antibody for 1 h. After washing twice using Tris-buffered saline-Tween (TBST) buffer and three times using TBS buffer, the membrane was incubated again for 1 h with the secondary antibody in 5 ml horseradish peroxidase (HRP)-rabbit anti-mouse IgG solution. The membrane was washed twice using TBST buffer and three times using TBS buffer. The membrane was stained with TrueBlue Peroxidase Substrate for visualization.

### Rice transformation

The genetic complementation and *Bph32* promoter-GUS fusion constructs were transformed into the *Agrobacterium tumefaciens* strain EHA105, and were then transformed into the receptor Kasalath (Ka). Plants regenerated from hygromycin-resistant calli (T_0_ plants) were self-pollinated to produce T_1_ and T_2_ seeds. The genotypes of each transgenic plant and their progenies were examined by PCR amplification using gene-specific primers (Table S6). Transgenic plants carrying *Bph32* were evaluated for BPH resistance using the above-described methods. GUS activity was detected in transgenic plants by histochemical assay^72^,^73^. The experiment was repeated three times.

### Evaluation of BPH resistance

BPH resistance was evaluated using the six-scale standard scoring system described by Heinrichs *et al.*^74^: 0 = no damage; 1 = very slight damage; 3 = first and second leaves partially yellowing; 5 = pronounced yellowing and stunting; 7 = mostly wilting, the plant still alive; and 9 = the plant completely wilted or dead. Five bioassay methods were used to evaluate BPH resistance in this study. Each experiment was repeated at least twice.

The modified standard seedbox screening method was used to measure the levels of resistance of the donor Ptb33, the susceptible TN1, the transgenic T_2:3_ plants, the receptor Kasalath (Ka), the introgression line 195B and its hybrid at the seedling stage under greenhouse conditions. Pre-germinated test seeds were soaked in Petri dishes (Ф10 cm) containing tissue paper to ensure that all seedlings were at the same growth stage before BPH infestation. Three days after soaking, the germinating seeds of each material were sown in a 40 × 20 × 2 cm seedbox at a row spacing of 20 × 4 cm with approximately 20 to 30 seeds in each row. At the third-leaf stage, the seedlings were infested with 2^nd^ to 3^rd^ instar BPH nymphs at a density of 8 to 10 insects per seedling. When all TN1 plants had died, the degrees of seedling damage in the varieties or lines were recorded. Each experiment was replicated three times.

Modified adult plant screening was used to evaluate the BPH resistance of the transgenic T_2:3_, Ka, 195B and 163B lines according to Suh *et al.*^75^. Three seedlings at the fourth-leaf stage of the transgenic T_2:3_, Ka, 195B and 163B lines were transplanted into 25-cm diameter plastic pots containing pulverized soil with compound fertilizer (15-13-12, N-P_2_O_5_-K_2_O) in three replicates. At the heading stage, each adult rice plant was infested with 2^nd^ to 3^rd^ instar nymphs at a density of 200 to 210 insects per plant. The transgenic T_2:3_ and 195B plants were evaluated based on the degree of damage in the susceptible Ka and 163B control plants, and the plants were recorded as resistant or susceptible once the susceptible controls had died.

In the seedling test, the BPH survival rate was determined to assess the reaction to BPH preference for 195B and 163B. To ensure that all seedlings were at the same growth stage, the seeds were pre-germinated on Petri dishes containing tissue paper. After seven days, the germinating seeds were sown into 15 × 12 cm plastic pots at the rate of one seed per pot. Ten pots were replicated for each treatment. Thirty days after transplanting, the plants were trimmed to one tiller. Then, each plant was infested by 20 2^nd^ instar nymphs and each pot was enclosed in a nylon mesh cage. The number of BPH on each plant was recorded at 1, 2, 3, 4, 5, 6, 7, 8, 9, 10, 11, 12, and 13 days post-infestation.

The honeydew excretion test, as a measure of feeding rate, was conducted to determine the phenotypic reaction of the plants to BPH feeding as described in Heinrichs *et al.*^74^. The specific steps used were as follows: seeds were first pregerminated on Petri dishes containing tissue paper for seven days. Three germinating seeds of each material were transplanted into one plastic pot, which was filled with clay as the medium. Ten plastic pots were replicated for each material. The tillers and pollutants were removed from the rice plants 30 days after the rice seeds were sown. Single tillers of the 30-day plants were transferred into 10-cm-diameter plastic cups equipped with honeydew-deposit chambers, which formed a semi-circular dome-like cover and a flat cover, at the rate of one plant per pot. Ten pots were replicated for each treatment. The base of each plant was encircled by a filter paper disk containing 0.02% bromocresol green. Female 4^th^ instar nymphs were starved for 2 h in a nylon mesh cage containing a moist filter paper and were transferred to the chambers (one nymph in each chamber), and the filter papers were collected 24 h later. This process was repeated three times, and the total area of blue-green spots was measured using the Image-Pro Plus 6 program.

A modified bulk seedling screening was used to evaluate the BPH resistance of 119 varieties of cultivated rice, rice accessions, and the 115 F_2:3_ lines of a population derived from a cross between 195B and 106B (susceptible) at the seedling stage under greenhouse conditions. Approximately 20 to 30 seeds of each material were pre-germinated in small nylon mesh bags for three days and were then sown (8 × 12 cm) in 100 cm × 150 cm pots. Each material was replicated twice. At the third-leaf stage, the seedlings were infested with 2^nd^ and 3^rd^ instar nymphs at a density of 8 to 10 insects per seedling. Each pot was then covered with a 100 × 150 cm nylon mesh cage. When all of the TN1 plants had died, the degrees of seedling damage in the other varieties or accessions were recorded.

### Sequence variation analysis

The exons of *Bph32* from 123 varieties of cultivated rice and rice accessions were obtained by PCR amplification and DNA sequencing using gene-specific primers (Table S6). The DNA sequences were translated using the Fgenesh gene-finder^38^ at http://linux1.softberry.com/berry.phtml?topic=fgenesh&group=programs&subgroup=gfind. Homologous proteins of *Bph32* were screened by BLAST searching at http://www.uniprot.org/blast/. Phylogenetic analysis was performed using the Clustal Omega program^76^,^77^,^78^ (http://www.uniprot.org/align/ or http://www.ebi.ac.uk/Tools/msa/clustalo/). The phylogenetic tree was built using Mega 6.05^79^.

### Genotypic analysis of line 195B

Four hundred twenty-six SSR markers that were distributed evenly on 12 rice chromosomes were chosen. The SSR primers and the InDel marker primers Bp3-In1and Bp3-In2 for *Bph3* and Bp26-INd for *BPH26* (Table S6) were synthesized by Sangon Biotech (Shanghai) Co., Ltd. The genetic characterization of 195B was analysed using these SSR and InDel markers. A genetic linkage map of 195B was constructed using the Mapdraw program^80^ based on the results of PCR amplification and the physical genetic distance of these SSR markers as reported by http://www.gramene.org/.

### RNA isolation and qPCR analysis

Pre-germinated seeds of 195B and 163B were sown in 15-cm- diameter pots at the density of one seed per pot. Each plant was infested with 20 BPHs 45 days after sowing and each pot was then covered with a nylon mesh cage. The leaves and sheaths of each plant were collected and frozen immediately in liquid nitrogen at 0, 2, 4, 8, 24 and 48 h after infestation. All treatments, each with three biological replicates, were terminated at the same time. At the heading stage, the flag leaves, culms, sheaths, and roots of 195B and 163B (each line with three biological replicates) were collected and immediately frozen in liquid nitrogen before total RNA isolation. Total RNA was extracted using a TaKaRa MiniBEST Plant RNA Extraction Kit according to the manufacturer’s instructions. Total RNA was then converted into first-strand cDNA using an AMV First Strand cDNA Synthesis Kit following the manufacturer’s instructions. Quantitative RT-PCR was performed using a LightCycler480 Software system (Roche) using the primers listed in Table S6 for the expression analysis of *Bph32*. The RT-PCR data were analyzed using the 2^−ΔΔC^^T^ method^81^.

## Additional Information

**How to cite this article**: Ren, J. *et al.* *Bph32*, a novel gene encoding an unknown SCR domain-containing protein, confers resistance against the brown planthopper in rice. *Sci. Rep.* **6**, 37645; doi: 10.1038/srep37645 (2016).

**Publisher’s note:** Springer Nature remains neutral with regard to jurisdictional claims in published maps and institutional affiliations.

## Supplementary Material

Supplementary Figure S1

Supplementary Figure S2

Supplementary Figure S3

Supplementary Figure S4

Supplementary Table S1

Supplementary Table S2

Supplementary Table S3

Supplementary Table S4

Supplementary Table S5

Supplementary Table S6
