# Supplementary material for: Bph32, a novel gene encoding an unknown SCR domain-containing protein, confers resistance against the brown planthopper in rice
Source: Sci Rep. 2016 Nov 23;6:37645. doi: 10.1038/srep37645 (PMC5120289; doi:10.1038/srep37645)
Supplement: Supplementary Figure S1 [file srep37645-s1.pdf]

Bph32, a novel gene encoding an unknown SCR domain-containing protein confers resistance against the brown planthopper in rice

Juansheng Ren[1]*, Fangyuan Gao[1]*, Xianting Wu[1]*, Xianjun Lu[1], Lihua Zeng[3],Jianqun Lv[1], Xiangwen Su[1],Hong Luo[2],and Guangjun Ren[1]**

[1]Crop Research Institute, Sichuan Academy of Agricultural Sciences, Chengdu, 610066, P.R. China

[2]Department of Genetics and Biochemistry, Clemson University,110 Biosystems Research Complex, Clemson, SC 29634-0318,USA

[3]Sichuan Normal University, Chengdu, 610066, P.R. China

*These authors contributed equally to the work.

**Corresponding author e-mail: guangjun61@sina.com

```
Ptb33           ATGGCAGCGATGATCGGGACGCTGGCCCTGCTCGCCGTGGGCTGCTCCGTGACCGTGGTC
IR60            ATGGCAGCGATGATCGGGACGCTGGCCCTGCTCGCCGTGGGCTGCTCCGTGACCGTGGTC
IR70            ATGGCAGCGATGATCGGGACGCTGGCCCTGCTCGCCGTGGGCTGCTCCGTGACCGTGGTC
195B            ATGGCAGCGATGATCGGGACGCTGGCCCTGCTCGCCGTGGGCTGCTCCGTGACCGTGGTC
121216          ATGGCAGCGATGATCGGGACGCTGGCCCTGCTCGCCGTGGGCTGCTCCGTGACCGTGGTC
TN1             ATGGCAGCGATGATCGGGACGCTGGCCCTGCTCGCCGTGGCCTGCTCCGTGACCGTGGTC
Nipponbare      ATGGCAGCGATGATCGGGACGCTGGCCCTGCTCGCCGTGGCCTGCTCCGTGACCGTGGTC
9311            ATGGCAGCGATGATCGGGACGCTGGCCCTGCTCGCCGTGGCCTGCTCCGTGACCGTGGTC
                **************************************** *******************

Ptb33           CTCAGCCCCGCGCACCTCGTCTTCGGCGCCCGCGTGAGGGAGGACTACTACTCCGGCAG-
IR60            CTCAGCCCCGCGCACCTCGTCTTCGGCGCCCGCGTGAGGGAGGACTACTACTCCGGCAG-
IR70            CTCAGCCCCGCGCACCTCGTCTTCGGCGCCCGCGTGAGGGAGGACTACTACTCCGGCAG-
195B            CTCAGCCCCGCGCACCTCGTCTTCGGCGCCCGCGTGAGGGAGGACTACTACTCCGGCAG-
121216          CTCAGCCCCGCGCACCTCGTCTTCGGCGCCCGCGTGAGGGAGGACTACTACTCCGGCAG-
TN1             CTCAGCCCCGCGCACCTCGTCTTCGGCGCCCTCGTGAGGGAGGACCACTACTACTACAAC
Nipponbare      CTCAGCCCCGCGCACCTCGTCTTCGGCGCCCTCGTGAGGGAGGACCACTACTACTACAAC
9311            CTCAGCCCCGCGCACCTCGTCTTCGGCGCCCTCGTGAGGGAGGACCACTACTACTACAAC
                ******************************* ************* ****** *  **

Ptb33           -----GACGCCGGAGCGGCAGATCAACGTCACCATCACCGCCAACAACACCAGCAAGCAC
IR60            -----GACGCCGGAGCGGCAGATCAACGTCACCATCACCGCCAACAACACCAGCAAGCAC
IR70            -----GACGCCGGAGCGGCAGATCAACGTCACCATCACCGCCAACAACACCAGCAAGCAC
195B            -----GACGCCGGAGCGGCAGATCAACGTCACCATCACCGCCAACAACACCAGCAAGCAC
121216          -----GACGCCGGAGCGGCAGATCAACGTCACCATCACCGCCAACAACACCAGCAAGCAC
TN1             AGGACGGCGCCGGAGCGGCAGATCAACGTCACCATCACCGCCAACAACACCAGCAAGCAC
Nipponbare      AGGACGGCGCCGGAGCGGCAGATCAACGTCACCATCACCGCCAACAACACCAGCAAGCAC
9311            AGGACGGCGCCGGAGCGGCAGATCAACGTCACCATCACCGCCAACAACACCAGCAAGCAC
                     * *****************************************************

Ptb33           GCCAAGGTGCGGTACCTGTCCATGAAGACCGAGGTGTGGCTGGACGACAAGGACTGGGTT
IR60            GCCAAGGTGCGGTACCTGTCCATGAAGACCGAGGTGTGGCTGGACGACAAGGACTGGGTT
IR70            GCCAAGGTGCGGTACCTGTCCATGAAGACCGAGGTGTGGCTGGACGACAAGGACTGGGTT
195B            GCCAAGGTGCGGTACCTGTCCATGAAGACCGAGGTGTGGCTGGACGACAAGGACTGGGTT
121216          GCCAAGGTGCGGTACCTGTCCATGAAGACCGAGGTGTGGCTGGACGACAAGGACTGGGTT
TN1             GCCAAGGTGCGGTACCTGTCCATGAAGACCGAGGTGTGGCTGGACGACAAGGACTGGGTT
Nipponbare      GCCAAGGTGCGGTACCTGTCCATGAAGACCGAGGTGTGGCTGGACGACAAGGACTGGGTT
9311            GCCAAGGTGCGGTACCTGTCCATGAAGACCGAGGTGTGGCTGGACGACAAGGACTGGGTT
                ************************************************************

Ptb33           CCGGTGGACCTGGGCACCGACAACAAGACCTCGAATCAATTCCGCACGTGGTGGCAGCCG
IR60            CCGGTGGACCTGGGCACCGACAACAAGACCTCGAATCAATTCCGCACGTGGTGGCAGCCG
IR70            CCGGTGGACCTGGGCACCGACAACAAGACCTCGAATCAATTCCGCACGTGGTGGCAGCCG
195B            CCGGTGGACCTGGGCACCGACAACAAGACCTCGAATCAATTCCGCACGTGGTGGCAGCCG
121216          CCGGTGGACCTGGGCACCGACAACAAGACCTCGAATCAATTCCGCACGTGGTGGCAGCCG
TN1             CCGGTGGACCTGGGCACCGACAACAAGACCTCCAATCAATTCCGCACGTGGTGGCAGCCG
Nipponbare      CCGGTGGACCTGGGCACCGACAACAAGACCTCCAATCAATTCCGCACGTGGTGGCAGCCG
9311            CCGGTGGACCTGGGCACCGACAACAAGACCTCCAATCAATTCCGCACGTGGTGGCAGCCG
                ******************************** ***************************

Ptb33           CCGAACAACTCCACGCAGTTCACGGCGAGGGTCAACGTCCTGGAGACGTATGGGCTGCCT
IR60            CCGAACAACTCCACGCAGTTCACGGCGAGGGTCAACGTCCTGGAGACGTATGGGCTGCCT
IR70            CCGAACAACTCCACGCAGTTCACGGCGAGGGTCAACGTCCTGGAGACGTATGGGCTGCCT
195B            CCGAACAACTCCACGCAGTTCACGGCGAGGGTCAACGTCCTGGAGACGTATGGGCTGCCT
121216          CCGAACAACTCCACGCAGTTCACGGCGAGGGTCAACGTCCTGGAGACGTATGGGCTGCCT
TN1             CCGGACAGCTCCACGCAGCTCACGGCGGGGGTCAACGTCCTGGAGACGTATGGGCTGCCT
Nipponbare      CCGGACAGCTCCACGCAGCTCACGGCGGGGGTCAACGTCCTGGAGACGTATGGGCTGCCT
9311            CCGGACAGCTCCACGCAGCTCACGGCGGGGGTCAACGTCCTGGAGACGTATGGGCTGCCT
                *** *** ********** ******** ********************************
```

Figure S1a. Comparison of nucleotide sequences in the exon of *Bph32* and its alleles from other rice varieties.

```
Ptb33          TACTACACGGTTGTGATCAAAACCCAGGTGCAGTTCAGGTACGGCCCTGCTCACACAAGG
IR60           TACTACACGGTTGTGATCAAAACCCAGGTGCAGTTCAGGTACGGCCCTGCTCACACAAGG
IR70           TACTACACGGTTGTGATCAAAACCCAGGTGCAGTTCAGGTACGGCCCTGCTCACACAAGG
195B           TACTACACGGTTGTGATCAAAACCCAGGTGCAGTTCAGGTACGGCCCTGCTCACACAAGG
121216         TACTACACGGTTGTGATCAAAACCCAGGTGCAGTTCAGGTACGGCCCTGCTCACACAAGG
TN1            GACTACACGGTTGTGATCAAAACCCAGGTGCAGTTCAGGTACGGCCCTGCTCACACAAGG
Nipponbare     GACTACACGGTTGTGATCAAAACCCAGGTGCAGTTCAGGTACGGCCCTGCTCACACAAGG
9311           GACTACACGGTTGTGATCAAAACCCAGGTGCAGTTCAGGTACGGCCCTGCTCACACAAGG
                ***********************************************************

Ptb33          CTCTACAGCATCATCGTCACCTGCCCCTCCAACACCAACTTATCATCATGGGGCAACAAT
IR60           CTCTACAGCATCATCGTCACCTGCCCCTCCAACACCAACTTATCATCATGGGGCAACAAT
IR70           CTCTACAGCATCATCGTCACCTGCCCCTCCAACACCAACTTATCATCATGGGGCAACAAT
195B           CTCTACAGCATCATCGTCACCTGCCCCTCCAACACCAACTTATCATCATGGGGCAACAAT
121216         CTCTACAGCATCATCGTCACCTGCCCCTCCAACACCAACTTATCATCATGGGGCAACAAT
TN1            CTCTACAGCATCATCGTCACCTGCCCCTGCAACACCAACTTAACACGGTACTACTACGAC
Nipponbare     CTCTACAGCATCATCGTCACCTGCCCCTGCAACACCAACTTAACACGGTACTACTACGAC
9311           CTCTACAGCATCATCGTCACCTGCCCCTGCAACACCAACTTAACACGGTACTACTACGAC
               **************************** ************* **   *    * ** *

Ptb33          GTTC---CTGATGAGGACCACTATTACTCCATCAACGACGTCTGTACCTATTAG
IR60           GTTC---CTGATGAGGACCACTATTACTCCATCAACGACGTCTGTACCTATTAG
IR70           GTTC---CTGATGAGGACCACTATTACTCCATCAACGACGTCTGTACCTATTAG
195B           GTTC---CTGATGAGGACCACTATTACTCCATCAACGACGTCTGTACCTATTAG
121216         GTTC---CTGATGAGGACCACTATTACTCCATCAACGACGTCTGTACCTATTAG
TN1            AGTAAAACTGATGTGGGCCACCTTTACTTCATCAACGACGTCTGTACCTATTAG
Nipponbare     AGTAAAACTGATGTGGGCCACCTTTACTTCATCAACGACGTCTGTACCTATTAG
9311           AGTAAAACTGATGTGGGCCACCTTTACTTCATCAACGACGTCTGTACCTATTAG
                 *    ****** ** ****  ***** *************************
```

Figure S1a. cont.

```
Ptb33          ATCACGAACTGCTTGCCGCTCACCTTTCCTCCGCCCCACCGCCGACATGCACCGCCGCAG
TN1            ATCACCAACTGCTTGCCGCTCACCTTTCCTCCGCCCCACCGCCGACATGCACCGCCGCAG
               ***** ******************************************************

Ptb33          CTCGTACTCCTCCTACTCCAAGCCATATATATTTAATTATACTCCAGTACTGGATACGTA
TN1            CTCGTACTCCTCCTACTCCAAGCCATATATATTTAATTATACTCCAGTACTGGATACGTA
               ************************************************************

Ptb33          CTCCTACTACAACTCCCTCAACTGATGACTGCTAGCGCTACATATATATAGGATAGGATA
TN1            CTCCTACTACAACTCCCTCAACTGATGACTGCTAGCGCTACATATATA--GGATAGGATA
               ************************************************  **********

Ptb33          TGATATCAAGGTCTTAAACCCTGTTTAGATGGGACTAAAACTTTTAAATCTCTATCATAT
TN1            TGATATCTAGGTCTTAAACCCTATTTAGATGGGACTAAAACTTTTAAATCCCTATCATAT
               ******* ************* ***************************** ********

Ptb33          TGGATATTGGATGTTTAGAAACTAATTATAAATATTAAACGTAGACTATTAATAAAACCC
TN1            TGGAT-------GTTTAGAAACTAATTATAAATATTAAACGTAGACTATTAATAAAACCC
               *****       ************************************************

Ptb33          ATCCATAATCTTGGACTAATTCGCGAGACGAATCTATTGAGTC--ATTAATCCATGATTA
TN1            ATCCATAATCTTGGACTAATTCGCGAGACGAATCTATTGAGTCTAATTAATCCATGATTA
               *******************************************  ***************

Ptb33          GCCTATGTGATGCTACAGTAAACATTCTCTAATTATAGATTAATTAGGCTTAAAATATTT
TN1            GCCTATGTGATGCTACAGTAAACATTCTCTAATTATGGATTAATTAGGCTTAAAATATTT
               ************************************ ***********************

Ptb33          GTCCCACGAATTAGCTTTCATTTATATAATTAGTTTTTAAGTAGTCTATATTTAATACT
TN1            GTCCCACGAATTAGCTTTCATTTATATAATTAGTTTTTAAGTAGTCTATATTTAATACT
               ************************************************************

Ptb33          CTAAATTAGTGTCTAAATACAAGAACTAAAGCTAAGTCCTTGGATCCAAACACCATCTTA
TN1            CTAAATTAGTGTCTAAATACAAGAACTAAAGCTAAGTCCTTGGATCCAAACACCACCTTA
               ******************************************************* ****

Ptb33          ATTAGGCCGATTGTGAAATTAATTAATTCAACTCCTCTCGAGTGTGTGATTTGTCGATCG
TN1            ATTAGGCCGATTGTGAAATTAATTAATTCAACTCCTCTCGAGTGTGTGATTTGTCGATCG
               ************************************************************

Ptb33          CATGCATGCAAGCACCATGATTACTGCACCACCCATCGATACTCCCCAGCTAACAAAAAG
TN1            CATGCATGCAAGCACCATGATTACTGCACCACCCATCGATACTCCCCAGCTAACAAAAAG
               ************************************************************

Ptb33          GAACAAAGGGGAGATTGTACGTACCATGCATTGCATGATTACTACACCATCCATCGATAC
TN1            GAACAAAGGGGAGATTGTACGTACCATGCATTGCATGATTACTACACCATCCATCGATAC
               ************************************************************

Ptb33          TCCCAGCTAACAAAGGACACAAAGGGGAAAGGTAATATCATCTTTTCCATTTATTTCTTG
TN1            TCCCAGCTAACAAAGGACACAAAGGGGAAAGGTAATATCATCTTT-CCATTTATTTCTTG
               ********************************************* **************

Ptb33          CTTGTGATC
TN1            CTTGTGATC
               *********
```

Figure S1b. Comparison of nucleotide sequences in the promoter region of *Bph32* and its alleles from TN1.
