# Supplementary material for: Bph32, a novel gene encoding an unknown SCR domain-containing protein, confers resistance against the brown planthopper in rice
Source: Sci Rep. 2016 Nov 23;6:37645. doi: 10.1038/srep37645 (PMC5120289; doi:10.1038/srep37645)
Supplement: Supplementary Figure S2 [file srep37645-s2.pdf]

Bph32, a novel gene encoding an unknown SCR domain-containing protein confers resistance against the brown planthopper in rice

Juansheng Ren[1]*, Fangyuan Gao[1]*, Xianting Wu[1]*, Xianjun Lu[1], Lihua Zeng[3], Jianqun Lv[1], Xiangwen Su[1], Hong Luo[2], and Guangjun Ren[1]**

[1]Crop Research Institute, Sichuan Academy of Agricultural Sciences, Chengdu, 610066, P.R. China

[2]Department of Genetics and Biochemistry, Clemson University,110 Biosystems Research Complex, Clemson, SC 29634-0318,USA

[3]Sichuan Normal University, Chengdu, 610066, P.R. China

*These authors contributed equally to the work.

**Corresponding author e-mail: guangjun61@sina.com

```
Ptb33                 1     MAAMIGTLALLAVGCSVTVVLSPAHLVFGARVRED--YYSGRTPERQINVTITANNTSKH      58
TN1                   1     MAAMIGTLALLAVACSVTVVLSPAHLVFGALVREDHYYYNRTAPERQINVTITANNTSKH      60
                            *************.**************** ****  **.  :*****************

Ptb33                 59    AKVRYLSMKTEVWLDDKDWVPVDLGTDNKTSNQFRTWWQPPNNSTQFTARVNVLETYGLP      118
TN1                   61    AKVRYLSMKTEVWLDDKDWVPVDLGTDNKTSNQFRTWWQPPDSSTQLTAGVNVLETYGLP      120
                            *****************************************:.***:** **********

Ptb33                 119   LSSSAPPPPPPPPGSSNDNKYYTVVIKTQVQFRYGPAHTRLYSIIVTCPSNTNLSSWG-N      177
TN1                   121   RSSSAPP---PPPGSSNDNKDYTVVIKTQVQFRYGPAHTRLYSIIVTCPCNTNLTRYYYD      177
                             ******   ********** ****************************.****: :  :

Ptb33                 178   NVPDEDHYYSINDVCTY                                                 194
TN1                   178   SKTDVGHLYFINDVCTY                                                 194
                            .  *  * * *******
```

Figure S2. Comparison of amino acid sequences in *Bph32* and its alleles from variety TN1.
