# Supplementary material for: Bph32, a novel gene encoding an unknown SCR domain-containing protein, confers resistance against the brown planthopper in rice
Source: Sci Rep. 2016 Nov 23;6:37645. doi: 10.1038/srep37645 (PMC5120289; doi:10.1038/srep37645)
Supplement: Supplementary Figure S3 [file srep37645-s3.pdf]

Bph32, a novel gene encoding an unknown SCR domain-containing protein confers resistance against the brown planthopper in rice

Juansheng Ren[1]*, Fangyuan Gao[1]*, Xianting Wu[1]*, Xianjun Lu[1], Lihua Zeng[3], Jianqun Lv[1], Xiangwen Su[1], Hong Luo[2], and Guangjun Ren[1]**

[1]Crop Research Institute, Sichuan Academy of Agricultural Sciences, Chengdu, 610066, P.R. China

[2]Department of Genetics and Biochemistry, Clemson University,110 Biosystems Research Complex, Clemson, SC 29634-0318,USA

[3]Sichuan Normal University, Chengdu, 610066, P.R. China

*These authors contributed equally to the work.

**Corresponding author e-mail: guangjun61@sina.com

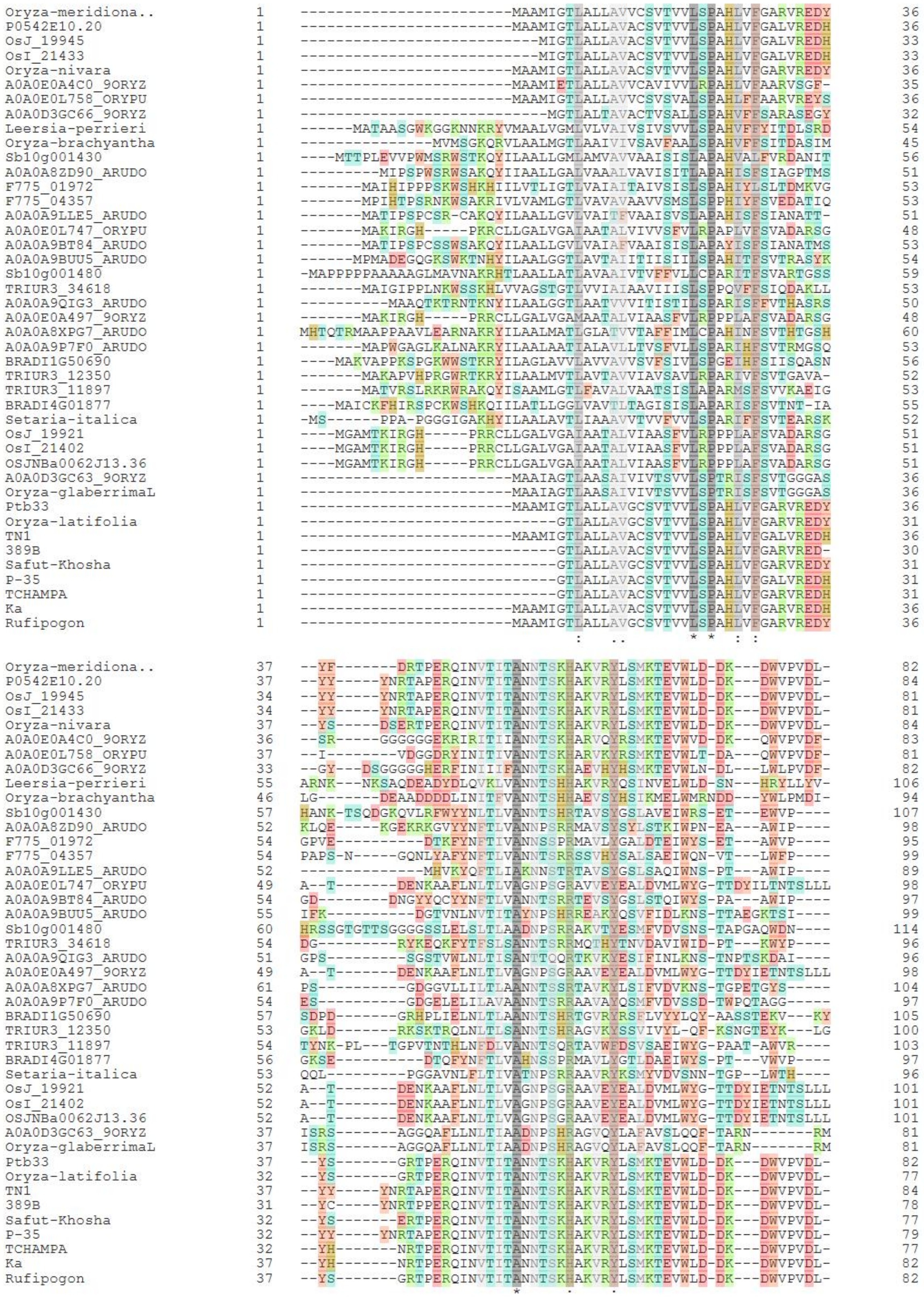

Figure S3. Comparison of amino acid sequences in homologs. Among 123 rice varieties and accessions, *Bph32* has eight alleles: the TN1 genotype:*bph32* (64), the Ptb33 genotype: *Bph32* (21), the 389B genotype: *bph32*^{389B} (15), the Kasalash genotype: *bph32*^{ka} (8), the *Oryza rufipogon* genotype: *bph32*^{Or}(7), the TCHAMPA genotype:*bph32*^{TC}(4), the P-35 genotype:*bph32*^{p} (2) and the

SafutKhosha genotype: *bph32^{SK}* (2), respectively. The numbers in brackets are the numbers of rice varieties and accessions with 100% identity in *Bph32* sequence. Or, *Oryza*; Sb, *sorghum bicolor*; ARUDO, *Arundo donax*; F775,*Aegilops tauschii*; TRIUA, *Triticum urartu*; BRADI, *Brachypodium distachyon*, respectively.

```
Oryza-meridiona..    83   -GTDNKTSNQFRTWWQPPDSSTQLTAGVNVL--E---TYGLPRSSSAPPPPP--------   128
P0542E10.20          85   -GTDNKTSNQFRTWWQPPDSSTQLTAGVNVL--E---TYGLPRSSSAPPPP---------   129
OsJ_19945            82   -GTDNKTSNQFRTWWQPPDSSTQLTAGVNVL--E---TYGLPRSSSAPPPP---------   126
OsI_21433            82   -GTDNKTSNQFRTWWQPPDSSTQLTAGVNVL--E---TYGLPRSSSAPPPP---------   126
Oryza-nivara         85   -GTDNKTSNQFRTWWQPPDSSTQLTAGVNVL--E---TYGRPRSSSAPP-----------   127
A0A0E0A4C0_9ORYZ     84   -DGKTSAQFSGDPWWQPPDNSTLLTARVHVL--E---ADEKKN---QPPPPG--------   126
A0A0E0L758_ORYPU     82   -DNYTTSVGFADLGWQPPANSTRLSAKKMIPPEG---TYELPRDNCKDS-----------   126
A0A0D3GC66_9ORYZ     83   -DRYAAA-GFNSLPWQPPANTTQISARFKAEGTTYKRSSPSPVDDNKPPPAP--------   132
Leersia-perrieri     107  -QD-----YLLSEQWQPPRNSTEYSATGPSY------FYGNS------------------   136
Oryza-brachyantha    95   -SNSVA----AGWGWQPAGNFTAYKVSSPLTSFN-----NESR--------H--------   128
Sb10g001430          108  -TEEVNTSAV-LPGWLPPGNTSLVKFGVEGGQSN---QETAPVKAQPPSSST--------   154
A0A0A8ZD90_ARUDO     99   -A-EVNTSAA-LPGWQPPGKTTDVTVLAESWQYD---QTTDAPKQ---------------   137
F775_01972           96   -AMVDLSVLQ-DGRRQAPGNVAHINVSAEYWQSE---QASGNKAPDQA------------   138
F775_04357           100  -A-EVDTPAV-VGKQQPPGGLTRVRGWAEFYEYD---RATKPSD----------------   137
A0A0A9LLE5_ARUDO     90   -A-EVNTTAM-LPEWQPPRNATNVAVWAEDGQYN---EAANATANAT-------------   130
A0A0E0L747_ORYPU     99   AADDG-TTAAAALLLQPPRNATAVEVTARTLDDR---FVGEI------------------   136
A0A0A9BT84_ARUDO     98   -A-EVN--TM-LPEWQPPRNATDVAIGAEYGQYN---EAANATANTTDHRQL--------   141
A0A0A9BUU5_ARUDO     100  -HANI-PDGTFPTSYLRGPNVTLINASVLLVDL------DG-------------------   132
Sb10g001480          115  -WVRA-TVTTRMPLRQTGRTAAINVTVPLVDDAP---WIQA-------------------   150
TRIUR3_34618         97   -AEVNMSGFQFQQYVQPPRNVTKVPWRAQWAQYD---NPTEGG-----------------   135
A0A0A9QIG3_ARUDO     97   -PAEV-DSGVLPKEYVTSPSVTNINASALLVGAA---IIES-------------------   132
A0A0E0A497_9ORYZ     99   ---A--AADEAALLLQPPRNATAVEVTARTLDDR---FVQEI------------------   132
A0A0A8XPG7_ARUDO     105  --VKA-DLTTDMPLSQRPASVANVNARASLVDTP---WTEG-------------------   139
A0A0A9P7F0_ARUDO     98   ------NVTTMLPLLQPTSNV-----TTIVVIVP---GADA-------------------   124
BRADI1G50690         106  STPAK-VIRAPPISQQPPLSTAEVEVSASLSMDE---PAMS-------------------   142
TRIUR3_12350         101  AG-----PASPPWHWQPPGATVSMSQSGTLFG-----TALD-------------------   131
TRIUR3_11897         104  -TKD----AGLPAGWQPPRSVISINVSADYGATR---AATNPRGLYPPPPPSFSLLPSSV   155
BRADI4G01877         98   -ALVDRTTAL-PDGRTPPDSETQMNVCAEYWQSK---QVVPTTTNNNNNQQQ--------   144
Setaria-italica      97   -WLKA-DVT-PMPLDQPTKSETRINAMVTLVTRS---QVED-------------------   131
OsJ_19921            102  VGLA--AADEAALLLQPPRNATAVEVTARTLDDR---FVQEI------------------   138
OsI_21402            102  VGLA--AADEAALLLQPPRNATAVEVTARTLDDR---FVQEI------------------   138
OSJNBa0062J13.36     102  VGLA--AADEAALLLQPPRNATAVEVTARTLDDR---FVQEI------------------   138
A0A0D3GC63_9ORYZ     82   ESVEA-TVQDGVPFYQPPASSRNVLVTASLVNKN---FFGAS------------------   119
Oryza-glaberrimaL    82   ESVEA-TVQDGVPFYQPPASSRNVLVTASLVNKN---FFGAS------------------   119
Ptb33                83   -GTDNKTSNQFRTWWQPPNNSTQFTARVNVL--E---TYGLPLSSSAPPPPP--------   128
Oryza-latifolia      78   -GTDNKTSNQFRTWWQPPNNSTQFTARVNVL--E---TYGLPLSSSAPPPPP--------   123
TN1                  85   -GTDNKTSNQFRTWWQPPDSSTQLTAGVNVL--E---TYGLPRSSSAPPPP---------   129
389B                 79   -GTDNKTSNQFRTWWQPPDNSTRLTAGVNVL--E---TYGRPRSSSAPPPP---------   123
Safut-Khosha         78   -GTDNKTSNQFRTWWQPPDSSTQLTAGVNVL--E---TYGRSRSSSAPP-----------   120
P-35                 80   -GTDNKTSNQFRTWWQPPDNSTQLTAAVNVL--E---TYGRPRSSLAPPPP---------   124
TCHAMPA              78   -GTDNKTSNQFRTWWQPPDNSTQLTAAVNVL--E---TYGRPQSSSAPPP-P--------   122
Ka                   83   -GTDNKTSNQFRTWWQPPDSSTQLTAGVNVL--E---TYGRPRSSLAPP-----------   125
Rufipogon            83   -GTDNKTSNQFRTWWQPPNNSTQFTARVNVL--E---TYGLPLSSSAPPPPP--------   128

Oryza-meridiona..    129  ------P---------------------PPGSSNDNKDYTVVIKTQVQFRYGP-AHTR--   158
P0542E10.20          130  -----------------------------PGSSNDNKDYTVVIKTQVQFRYGP-AHTR--   157
OsJ_19945            127  -----------------------------PGSSNDNKDYTVVIKTQVQFRYGP-AHTR--   154
OsI_21433            127  -----------------------------PGSSNDNKDYTVVIKTQVQFRYGP-AHTR--   154
Oryza-nivara         128  ------P---------------------PPGSSNDNKDYTVVIKTQVQFRYGP-AYTR--   157
A0A0E0A4C0_9ORYZ     127  ------K---LAGDT------STTTAAGASPSPSDNKEYTVVIKTQVQFSYGP-ARTR--   168
A0A0E0L758_ORYPU     127  ------------GG-----------NDQCTKNNSSSKEHTVVIKTQVQFRYGP-VPTR--   160
A0A0D3GC66_9ORYZ     133  ------P---AAAAAPGAAGNEDANTRNGTGVVHGNATYRVVIRTQVRFRYGP-ARTR--   180
Leersia-perrieri     137  -----------------------THDDYGVTYNNRSRYPMLLIETVVQFRYGP-SRTR--   170
Oryza-brachyantha    129  ------E---ITGG------GQQNGASSGDDDDEEQRHPVVVIRAHLRFRYGQYARTR--   171
Sb10g001430          155  P----------AQGASGNAAVGNDSNAAAAGGVPKWPDCRVVVEAKVWFRFA-GVPTL--   201
A0A0A8ZD90_ARUDO     138  ------------------------------DIKVDNNNCTVAVEAKVRFKFG-LARTM--   164
F775_01972           139  -----------------SSGGIGSPPPPPATNNTDWSNCTVVVMAKVWFKAG-GISTR--   178
F775_04357           138  ------------------------------IVVGDWPNSTVVVVAKVWFKFS-LARTR--   164
A0A0A9LLE5_ARUDO     131  ----------------------------VDGTAEQSTDCRVVVEAKVWFKYG-LAT-R--   158
A0A0E0L747_ORYPU     137  -----------------------------AGEESRAGPFNVAVAAQVRFKV-GMVYSR--   164
A0A0A9BT84_ARUDO     142  GTVEKSVFDCRRVALEANATANATVDDCQLGTVEKSVDCRVVVEAKVWFKYG-LTT-R--   197
A0A0A9BUU5_ARUDO     133  ----------------------------AYTRRLNGSGLVVVVRALVRFKV-GVTPTR--   161
Sb10g001480          151  ----------------------------FTGG---G-RFSVMVTAQARFRV-GVAWTR--   175
TRIUR3_34618         136  -----------------------------GGSAGHWPNCTVVVMAKINHANL-HMKNH--   163
A0A0A9QIG3_ARUDO     133  ----------------------------FADHPMNNSGLTVVVMAQVHFKI-GVVRTR--   161
A0A0E0A497_9ORYZ     133  ----------------------------VAGEGRRTGPFNVAVAAQVRFKVAGVVYTR--   162
A0A0A8XPG7_ARUDO     140  ----------------------------FTGNKTCN-TFAVIVAAVARFKV-GIARTR--   167
A0A0A9P7F0_ARUDO     125  ----------------------------ITGNKTG--NLTVIVTTVVWFKV-GIARTR--   151
BRADI1G50690         143  ----------------------------GGRRSNNNNIISVLLVSVVQFKAGPAY-WL--   171
TRIUR3_12350         132  ----------------------------IG-PSGRAPAIGVLVLAVVKYRVGPAAYTR--   160
TRIUR3_11897         156  HPREKPPWSLRLRGLFHAVVGDGTDLAAPQ------PFLESQARPTTWTPAEEKQRRRSS   209
BRADI4G01877         145  QQ----PL---PVG---QAPAISPPATRSTANNTDWSSCTVMVIAKVWFKAGSGISTR--   192
Setaria-italica      132  ----------------------------FTGNEMSH-SFRVMITTVVRFKV-GVSGTR--   159
OsJ_19921            139  ----------------------------VAGEGRRTGPFNVAVAAQVRFKVAGVVYTR--   168
OsI_21402            139  ----------------------------VAGEGRRTGPFNVAVAAQVRFKVAGVVYTR--   168
OSJNBa0062J13.36     139  ----------------------------VAGEGRRTGPFNVAVAAQVRFKVAGVVYTR--   168
A0A0D3GC63_9ORYZ     120  ----------------------------HGGGGGRGPPFTVVVKGQVRFKV-WLAYSR--   148
Oryza-glaberrimaL    120  ----------------------------HGGGGGRGPPFTVVVKGQVRFKV-WLAYSR--   148
Ptb33                129  ------P---------------------PPGSSNDNKYYTVVIKTQVQFRYGP-AHTR--   158
Oryza-latifolia      124  ------P---------------------PPGSSNDNKYYTVVIKTQVQF-----------   145
TN1                  130  -----------------------------PGSSNDNKDYTVVIKTQVQFRYGP-AHTR--   157
389B                 124  -----------------------------PPP-PGSMDYTVVIKTQVQF-----------   142
Safut-Khosha         121  ------P---------------------PPGSSNDNKDYTVVIKTQVQF-----------   142
P-35                 125  -----------------------------PPP-GSSNDYTVVIKTQVQFRYGP-AHTR--   151
TCHAMPA              123  ------S---------------------PPGSSNDNKDYTVVIKTQVQF-----------   144
Ka                   126  ------P---------------------PPGSSNDNKDYTVVIKTQVQFRYGP-AHTR--   155
Rufipogon            129  ------P---------------------PPGSSNDNKYYTVVIKTQVQFRYGP-AHTR--   158
```

Figure S3. Cont.

```
Oryza-meridiona..     159  LYSIIVTCPCN---------------------------TNL-SWVNYDNKTNADHY----  186
P0542E10.20           158  LYSIIVTCPCN---------------------------TNLTRY-YYDSKTDVGHL----  185
OsJ_19945             155  LYSIIVTCPCN---------------------------TNLTRY-YYDSKTDVGHL----  182
OsI_21433             155  LYSIIVTCPCN---------------------------TNLTRY-YYDSKTDVGHL----  182
Oryza-nivara          158  LYSIIVTCPCI---------------------------IVT-CLCTY-------------  176
A0A0E0A4C0_9ORYZ      169  FYNIIVTCPPS---------------------------ANANISSDYDTATY--VY----  195
A0A0E0L758_ORYPU      161  LYSILVTCPSV---------------------------AIS-------ESDYDDSI----  182
A0A0D3GC66_9ORYZ      181  LYSILVTCPSV---------------------------PIFDAADNNSTL----------  203
Leersia-perrieri      171  MYTVAVSCPSV---------------------------TVPWFFGEAEG--GGDHLNNT-  200
Oryza-brachyantha     172  LYRVVVSCLSV---------------------------NFIIFRPDGT---YD-YFLSP-  199
Sb10g001430           202  PYTVRVSCFPV---------------------------NFLDGT----------------  218
A0A0A8ZD90_ARUDO      165  GYTVRATCLHV---------------------------NFIKNA----------------  181
F775_01972            179  SYNIRASCSLV---------------------------NFDRAD----------------  195
F775_04357            165  PYNVRVTCAPV---------------------------NFRDKT----------------  181
A0A0A9LLE5_ARUDO      159  PYTVTASCLHV---------------------------NFFNGT----------------  175
A0A0E0L747_ORYPU      165  PYNVRVSCSDV---------------------------YFAVVA------------YNKS  185
A0A0A9BT84_ARUDO      198  PYTVTASCWHV---------------------------NFFNGT----------------  214
A0A0A9BUU5_ARUDO      162  LYEMKVSCPGV---------------------------LFSIEGSNYSA-----H-----  184
Sb10g001480           176  LYDIKVSCSPV---------------------------TFFPAKARPAD-----D--AAT  201
TRIUR3_34618          164  SY----RCWGFGPSLRKEKVSNSRYKDSDLIFVKEAKAKFYEGN----------------  203
A0A0A9QIG3_ARUDO      162  LYGIKVYCHGV---------------------------HFTDSSKSSAL-----S-----  184
A0A0E0A497_9ORYZ      163  PYNVRVSCSDV---------------------------YFVVAD------------NKSA  183
A0A0A8XPG7_ARUDO      168  LYDIKVSCGPV---------------------------SFFPAAADHSG-----A-----  190
A0A0A9P7F0_ARUDO      152  LYDIKVTCQPV---------------------------SFFPNASKKPY-----H-----  174
BRADI1G50690          172  PYQVRVQCNPI----------------------------DSLF-------------SGSL  190
TRIUR3_12350          161  PYDVRVLGGGG---------------------------AVAGLLQRGGS-----GFAGLR  188
TRIUR3_11897          210  AFTRRTAM-SL--------------DTSSMLRPKAPAHANTDRI----------------  238
BRADI4G01877          193  SYDVRASCSQL-------------------------------------------------  203
Setaria-italica       160  LYVIKVACGPL---------------------------DFFAKQSRNAV-----V-----  182
OsJ_19921             169  PYNVRVSCSDV---------------------------YFVVAD------------NKSA  189
OsI_21402             169  PYNVRVSCSDV---------------------------YFVVAD------------NKSA  189
OSJNBa0062J13.36      169  PYNVRVSCSDV---------------------------YFVVAD------------NKSA  189
A0A0D3GC63_9ORYZ      149  PYDVAVECAPV---------------------------DVFSGA------------GGA-  168
Oryza-glaberrimaL     149  PYDVAVECAPV---------------------------DVFSGA------------GGA-  168
Ptb33                 159  LYSIIVTCPSN---------------------------TNLSSWGN--NVPDEDHY----  185
Oryza-latifolia       146  ------------------------------------------------------------  145
TN1                   158  LYSIIVTCPCN---------------------------TNLTRY-YYDSKTDVGHL----  185
389B                  143  ------------------------------------------------------------  142
Safut-Khosha          143  ------------------------------------------------------------  142
P-35                  152  LYSIIVTCPCN---------------------------TNLT------------------  166
TCHAMPA               145  ------------------------------------------------------------  144
Ka                    156  LYSIIVTCPCN---------------------------TNL-TRDYDDD-----------  176
Rufipogon             159  LYSIIVTCPSN---------------------------TNLSSWGNYNNVPDEDHY----  187

Oryza-meridiona..     187  ----YFIND-VCTY----------------------------------------------  195
P0542E10.20           186  ----YFIND-VCTY----------------------------------------------  194
OsJ_19945             183  ----YFIND-VCTY----------------------------------------------  191
OsI_21433             183  ----YFIND-VCTY----------------------------------------------  191
Oryza-nivara          177  ------------------------------------------------------------  176
A0A0E0A4C0_9ORYZ      196  ----SSRND-RCT-----------------------------------------------  203
A0A0E0L758_ORYPU      183  ----YSIND-FCSW----------------------------------------------  191
A0A0D3GC66_9ORYZ      204  --------N-ACKA----------------------------------------------  208
Leersia-perrieri      201  --IANYPPV-NCTA----------------------------------------------  211
Oryza-brachyantha     200  --SKATLPV-SCKA----------------------------------------------  210
Sb10g001430           219  -----TGPV-NCTG----------------------------------------------  226
A0A0A8ZD90_ARUDO      182  -----YFPV-PCLTA---------------------------------------------  190
F775_01972            196  -----A-IV-DCTHG---------------------------------------------  203
F775_04357            182  -----TYTA--CMD----------------------------------------------  188
A0A0A9LLE5_ARUDO      176  -----GVPV-VCGSS---------------------------------------------  184
A0A0E0L747_ORYPU      186  -AAATSTPI-DCHG----------------------------------------------  197
A0A0A9BT84_ARUDO      215  -----GVPV-TCVSS---------------------------------------------  223
A0A0A9BUU5_ARUDO      185  -----QRNF-NCSG----------------------------------------------  192
Sb10g001480           202  AAGASGLPV-RCV-----------------------------------------------  213
TRIUR3_34618          204  -----DHAIVSAVTSCYIQMVMFVRSVYPYAYNLTSTYASQKN-EGTMVATSVLMFILAV  257
A0A0A9QIG3_ARUDO      185  --------V-SCLD----------------------------------------------  189
A0A0E0A497_9ORYZ      184  A-AASSTPI-DCRG----------------------------------------------  195
A0A0A8XPG7_ARUDO      191  PAPAGLLPV-NCV-----------------------------------------------  202
A0A0A9P7F0_ARUDO      175  PAVICA------------------------------------------------------  180
BRADI1G50690          191  -----ATPI-NCTS----------------------------------------------  198
TRIUR3_12350          189  WARPGLGGL-VCHCGRVRLAPATM---------------LEAEASRTMVE-EVVPFDEAQ  231
TRIUR3_11897          239  -----GGPLGPTATGD---------GDWSV-GGARDKANLRGN-EAPLVATSVVMFVLAV  282
BRADI4G01877          204  ------------------------------------------------------------  203
Setaria-italica       183  ------------------------------------------------------------  182
OsJ_19921             190  AATASSTPI-DCHG----------------------------------------------  202
OsI_21402             190  AAAASSTPI-DCHG----------------------------------------------  202
OSJNBa0062J13.36      190  AAAASSTPI-DCHG----------------------------------------------  202
A0A0D3GC63_9ORYZ      169  KTSPKSTMV-KCIP----------------------------------------------  181
Oryza-glaberrimaL     169  KTSPKSTMV-KCIP----------------------------------------------  181
Ptb33                 186  ----YSIND-VCTY----------------------------------------------  194
Oryza-latifolia       146  ------------------------------------------------------------  145
TN1                   186  ----YFIND-VCTY----------------------------------------------  194
389B                  143  ------------------------------------------------------------  142
Safut-Khosha          143  ------------------------------------------------------------  142
P-35                  167  ------------------------------------------------------------  166
TCHAMPA               145  ------------------------------------------------------------  144
Ka                    177  ------------------------------------------------------------  176
Rufipogon             188  ----YSIND---------------------------------------------------  192
```

Figure S3. Cont.
