# Supplementary material for: Bph32, a novel gene encoding an unknown SCR domain-containing protein, confers resistance against the brown planthopper in rice
Source: Sci Rep. 2016 Nov 23;6:37645. doi: 10.1038/srep37645 (PMC5120289; doi:10.1038/srep37645)
Supplement: Supplementary Figure S4 [file srep37645-s4.pdf]

Bph32, a novel gene encoding an unknown SCR domain-containing protein confers resistance against the brown planthopper in rice

Juansheng Ren[1]*, Fangyuan Gao[1]*, Xianting Wu[1]*, Xianjun Lu[1], Lihua Zeng[3],Jianqun Lv[1], Xiangwen Su[1],Hong Luo[2],and Guangjun Ren[1]**

[1]Crop Research Institute, Sichuan Academy of Agricultural Sciences, Chengdu, 610066, P.R. China

[2]Department of Genetics and Biochemistry, Clemson University,110 Biosystems Research Complex, Clemson, SC 29634-0318,USA

[3]Sichuan Normal University, Chengdu, 610066, P.R. China

*These authors contributed equally to the work.

**Corresponding author e-mail: guangjun61@sina.com

>195B-12540 (*bph3)*

TGGTGACGAAGACCTTTCCATGACAAGTGTTAGGATAGCTCTATAACTACACAAGTTTGGCAGCTCACCAGC
TGTTATCAGGGGTAGGAAGCAAGAGCCTGAAGCAAAGAACAATGGTTGCTCTGCTACTCTTTCCCATGCTGT
TGCAGCTACTGTCCCCAACTTGTGCACAAACTCAGAAGAACATCACATTGGGCTCCACTTTGGCACCCCAGA
GTCCTGCCTCTTCATGGCTATCACCCTCTGGCGACTTCGCATTTGGCTTCCGACCTGTAGAGGGCAACACCTC
CTTCTACCTCATCGCCGTCTGGTTCAACAAGATTAGCGATAAGACAGTTGTCTGGTATGCCAAAAATACTGAC
CAAGATCCATCGATAGTAGAAGTGCCATCTGATTCTTTTCTCCAACTCACTAATGATGGTGCACTCTCACTGAA
AGACCGGTCTGGTCAGGAGGGCTGGAATCCCCAGGTTACCAGTGTGGCATATGCTAGCATGCGCGACACCG
GCAATTTTGTGCTCCTTGGTGCAGACGGCACGACAAAGTGGCAGACCTTTGACATGCCGTCAGACACCATC
TTGCCTACTCAGGTGATACCTTGCAACAAGACTCGCAACAAGTCACTCCGTGCCCGACTCGACATCGATGAC
TACTCCAGTGGTCGGTTTCTCCTTGATGTGCAAACTGATGGAAACCTTGCCCTGTATCTTGTTGCTGTTCCCTC
TGGATCCAAATACCAGCAATATTGGTCCACTGACACGACTGGAAATGGCTCAGAGTTGGTATTCAGTGAGAC
TGGGAAGGTATACTTTGCACTGACAGATGGTACACAGATCAACATCTCATCAGGTGCAGGAATTGGATCAAT
GGCAGACTACTTCCATCGCGCCACACTGGACCCTGACGGTGTTTTCCGGCAATATGTGTACCCGAAGAAAGC
GAATGCAGGCATCCTAGGTGGTGAGACATGGACAGCAGTGAGCATGCAGCCCCAGAATATCTGCCATGCCAT
AGTTTCAGATGTTGGCAGTGGCGTCTGTGGGTTCAACAGTTACTGCACCTTTGATGGCACCAGGAATCAGAT
TGCAAGCTGCCAATGCCCACCATGGTACAAGTTCTTCGATGAGCAGAAGAAGTACAAAGGGTGTAAGCAAG
ACTTTCAACCGCATAGCTGCGACTTGGATGAGGCAACGGCACTTGCTCAGTTTGAGCTGAGACCAATTTATG
GTGTGGATTGGCCACTGTCTGACTATGAGAAATATGAGCCCATAGGACAAGATGATTGTGGAAGGCTGTGTG
TGATCGACTGCTTCTGCGCCATGGCGGTGTACAATCAGAGCACCAGCACTTGCTGGAAGAAGAAGCTCCCT
TTGTCAAATGGGAATATGGCTGACTATGTCCAGAGGACCGTTCTTCTCAAGGTGCCAAGTAGCAATAGTTCG
CAATCCATGATCAGTACAAGCTCCAACAAATGGAAGAGGAACAGGAAGCACTGGGTCCTAGGGAGTTCTTT
GATTCTTGGAACCTCCATATTGGTGAACTTTGCACTCATCTCCATTTTCCTTTTTGGTACTTACTGTAGAATAGC
CACAAAAAAGAATATCCCATTGTCACAAGCATCAAGTAAATCCCAACTACCCTTGAAGACCTTCACTTACAAA
GAGCTGGAGAAGGCAACGGCTGGCTTCCATGAGATTCTAGGTGCCGGTGCCTCTGGTGTTGTGTACAAGG
GCCAGTTGGAGGATGAACTCaAGACCAACATTGCAGTGaAAAAGATTGACAAGCTCCAACCAGAAACCGA
GAAGGAGTTCATGGTAGAAGTCGAGACCATTGGGCAGACATtCCACAAGAATTTGgTTAGATTGCTTGGTTT
CTGTAATGAAGGAGCCGAAAGACTGCTAGTTTATGAGTTCATGACCAATGGCCCTCTCAACCGTCTTTTGTTC
GACAATAGTCGCCcACATTGgAACAcCCGAGTTCATATCGCGCTTGGGGTGGCAAGAGGTTTGCTCTACTTAC
ATGACGAATGTAGCAAGCAAATCATCCATTGTGACATAAAGCCGCAGAATATCCTTCTTGATGACAACCTTGT
GGCCAAGATTTCAGACTTTGGCCTAGCAAAGCTTCTTTTGACAAACCAAACAAGGACAAACACCGGTATTC
GGGGTACTCGAGGTTATGTTGCACCTGAGTGGTTCAAGAACATTGGGATCTCTACCAAGGTTGATGTTTATA
GCTTCGGAGTGATACTACTAGAGCTTGTTTGCTGTAGGAGGAATGTCGAGCTAGAGGTTGTCGATGAGGAA
CAGACGATAGTGACTTACTGGGCCAATGACTGCTACCGGTCTGGGAGGATTGATTTGCTGGTAAAGGGCGA
TGATGAGGCGATCTACAATATTAAGAAGGTGGAAAGATTTGTGACAGTGGCATTGTGGTGCCTTCAGGAGG
ACCCAAGCATGCGACCTAACATGCTAAAGGTGACACAAATGCTTGATGGTGCAGTTGCAATCCCCAGTCCTC
CTGATCCCTGTTCCTTCATCAGCTCACTTCCATAGTATCTCTTGCATGGTCAAATAAGTTTTCATATGCGTCAAA
ACAATAAGCATTTGGTGAATAGCTTTAGCTATATTCAGAAGGCAAATAGTTATTAATTCGGCATTGTACTGTAG
CAACTTTTAGATCTCCCATGTGTAGCTATAATTGAGTGGGATACACAACTGGTCCATCTCTTTGAGCTAGATAT
TTATCTATATCTGTTATGTAATAAAAAAAGGAATTATAGCTATACATTCAACTAATATTTCCTAGTAGAATTATAG
CTTCTAAAATAATTCATCGAATAATACAATGAATATCTTTTAATCCATATTTTGGAAATGAGAAGAGAAAATAG
AAGATGACAAACGTAGTAGGAGACTTCTTTTTTGTGTGTTTGGAATGTAATAAACGTGTTACTGAGACAGTA
GTTATAGTGTATGAGGGTTGTTCTGGATCGATGGCAAAACATATCCTAACAATGTTTTGATAGATTGCAAAGT
AAATATGATAAGTTCAGTTTGAACCGAGCGGTTGCCATCGGAAACCAGTTGAACAAAATTTTTCTAAACTTT

TTTTTACTCTTTTTTCAACTTGACTTGTTTGTTCAAGCGCTTCTAAATGTGGATACGACTTTTTGAAGAACTAC
GAAGATTGCACTCTAATGACTCGGCAACAACCTGATGATGTGAAAGGAAAAGGACCAATGTTTGTTGAGAC
ATACATACAACAACAGCTGAACGAAAAGCAAGTGGCAAACTCCACCAAAGGTGTAGCCAAGTTGAATGTTG
ATGCAGGTTTCAAATAGGACACAGGTGAAGCAGGCGCTGGGATAATAATCAGGGATTGTCGAGGCCTCGTG
CTACTGGCTTCCTGTAAGAAACTACCACGGTGCAGCTCAGCAATGCAAGCTGAAGCCTTGGTGTGTCTGGA
AGGTATCCGGCTTGCTACTGAATGGGTGAATATGCTACTTATCTTAGAATCAGATAATGCAAGTGTTGTCGCT
GAGTTAAACGCGACCAATGTCTCAAGAGCAGATTGGAGTGGCATAATTTCAGATGTTAAAGCGGCAATGTTA
TGTTTAGCTCAGGTGCAAGTGCACAGGATAAAGAGAGATTCTAATAAAATAGCTTATGTTTTAGCCCAACTG
GTAATGGGATCAGGAACTAATACGGTCTGGAGATTGTGTTCCCCTGCAGAAATTCTTGATCTCTTGAACCAG
GAATGTAACCCTCTGTTTTCTCATTAATGAAATCTCTCTTTACTTTCAAAAAAAATAAATGTGGATACGACTCA
CAAGCC

>195B-12560 (*bph3*)
CAGGTCAAAAAATTGTATAGAGAAACTTTTGGTTAGTCAAGTATGGAAGTGATCAAGACTAACAAATGCCGT
TCTCCAAGGGCTATAAGTTTTGTTGTCATCAAGTATGAGCAAAAGGTTGATCAATAGCAATGGCACCTATCCT
GTTCTTGCCCATCCTGCAAATCTTGCTAATATACTGCACGAAATCTGCCCAAGCTCAGCTAAACATCAGCATAG
GCTCCTCTTTGACACCCCAGGAGGTAAACAACTCATGGATTTCGCCCTCTTCTGATTTTGCATTTGGTTTTCG
GGCGGTAGATGGAAACTCCTCGTCATACCTGCTCGCTGTCTGGTTCAACAAGATCGCTGACAAGACTGTCAT
CTGGTACGCAAAGACTAGCTCCAATGGGCAAGATGATACAATACCGGTACAAGTCCAATCTGGCTCTGTCCT
CAAGCTCGCTGATGGAGCACTCTCTCTTCGTGATCCATCTGGCAATGAGGTATGGAATCCACGAGTCACTGAT
GTGGGCTATGCTAGAATGCTCAACACCGGTAATTTCAGGCTTTTAGGCACTGATGGCGCAACAAAGTGGGA
GTCCTTTGGTGACCCTTCTGATACCATCCTACCCACACAAGTGCTCCCACTAGGGACAGCACTCCACAGCCGT
CTCCTCGCCACAGACTATTCCAATGGCCGGTTTCAACTAAATGTTCAAGATGACGGTAATCTTGTGCTGTATCT
AGTTGCTGTACCTTCTGCATACTACCATGATCCATACTGGGCCAGTAACACAGTGGGGAATGGCTCACAGCT
GGTGTTCAATGAAACTGGAAGGATATACTTCACGCTGACTAATGGTTCACAGATAAACATCACTTCTGCAGG
AGTGGACTCTATGGGTGATTTCTTCCACCGTGCAACCCTTGATACAGATGGTGTGTTCCGGCAATATATTTACC
CAAAGAGCAAACAGGCCAGGAGCTTATGGCAAGAGCAATGGAGAGCGGTGGATGCACTTCCGGAGAACA
TCTGCCAGACAATTCAAACAAAGGTAGGCAGTGGTGCATGCGGCTTCAACAGTTACTGTACCTTTGATGGCA
CCAAGAACACAACAAATTGTCTATGCCCACAGAGGTACAAGTTCTTCGACAATGAGAGGACATACAAAGGT
GCAGGCCAGATTTTGAGCCACAAAGCTGTGATCTAGATGAGACAGCAGCAATGGTGCAGTATGAGATGACA
CCAATTGATCGCATTAATTGGCCTCTATCTGACTATGAGCAGTACAGCCCGATAGATGAGACCGAATGCCGAA
GGCTGTGCGTCATTGATTGCTTCTGCTCCGTTGCCGTGTTCAATAAACCCTCAAACACTTGTTATAAAAAGAA
GCTCCCTTTATCAAATGGGAATATGGATTCCAGTTTACAGGCGACAGTTCTTCTTAAGGTGCCTAGGAGCACC
AATTCACCATCCATGATCAGCAGCGGCTCCAGCAAATGGAAGAAGGACAAAAAGTATTGGATTCTTGGGAG
CTCATTGTTTTTTGGAAGCTCTGTATTGGTGAACTTTCTCCTAATCTTTGTTCTGCTTTTTGGTACTTATTGTAG
TATCACCTCGAGGAAGAAAACCCAGTTATCGCAACTACCCAGTAATTCTGGATTACCTTCAAAGATTTTCACT
TACAGAGAGCTGGAAAAGGCAACCGGTGGTTTCCATGAGGTACTTGGTACAGGAGCCTCAGGTATTGTCTA
CAAAGGACAGCTGCAAGATGAGTGTGGAACTAACATCGCAGTCAAGAAAATCGAAAAGCTTCAGCAGGAA
GCACAAAAGGAGTTCTTGGTGGAAGTCCAAACCATTGGGCAGACGTTTCACAGGAACTTAGTTAGACTGCT
TGGTTTTTGCAATGAGGGAACTGAAAAGCTGCTAGTGTATGAGTTCATGAGCAATGGCTCACTCAATACATT
CCTCTTCAACGATACTCATCCGCATTGGAGCCTCCGTGTTCAAGTAGCACTTGGGGTTTCACGAGGACTGCT
CTACCTACATGAGGAGTGTAATAAACAAATCATCCACTGCGATATGAAACCACAGAATATCCTTCTCGATGATA
ATTTTGTAGCAAAGATTTCAGATTTTGGTCTAGCAAAGCTTCTTCCGGTGAATCAGACACAAACAAACACCG
GCATTCGGGGTACTCGAGGATATGTTGCACCTGAGTGGTTCAAGAACATAGGGATTACTTCTAAGGTCGACG

TTTATAGCTTTGGAGTGATCCTGCTTGAGCTTGTGTGCTGCAGGAAGAATGTGGAATTAGAAGTTGCAGATG
AGGAGCAGACAATACTAACTTATTGGGCAAATGATTGCTACAGGTGTGGGAGGATTGACTTGCTGGTGGCG
GGCGATGACGAGGCAATCTTCAACATAAAAAAGGTGGAGCGCTTCGTTGCTGTGGCACTGTGGTGCCTCCA
GGAGGAGCCATCGATGCGGCCAACAATGCATAAGGTGATGCAGATGCTTGATGGAGCAGTACAAATCCCAA
CGCCTCCTGACCCTTCGTCCTATATCAGTTCACTCGCATGATAGAAGCATTCCTGTTAATAAACAAAAGATTGT
AATTTGAAGCAATAGCATTCTTTAGACTGATACTTCTTCGATAGGGCTTCGTGTTAAAGGCTTAAAGCATGATA
TGAGTCATGTTATTCAGGGCACTCATGCCTGCCTGCAACAAATAATGTTTACATACATATGAAAGATTATGATA
CATGAGCTCTGAGCCTCTGAATCTGTTTGAGTCAAACATTTTCTTACTGAAATAGATGACTTTAAATATGTTTG
ATCATGAAGTAATATTATCTCCTCTACAACCACATGATCCTGCAATTGGAAATTTGGAATCAAATAATCCTTGGC
TACTTCTTATATGTTCTTATACTGAAAGAGTGTACATATGTTCTTACCAGCATATTTCACATCTATAAAAAAAACA
CCTCCTTTTTTCTATTAGAAAGGAAAAGAACAGAAAATCACAAAAA

>195B-12580 (b*ph3*)
TTTTCTAGAGTTCTGATAATGAAATGTGAACTAATCTCCCTCTCATGTCTATCATATTGACAGCTTTGTAATTGC
ATTTGACAGGTTCAACCGTTGCCTCACAGGTCTCAAAAGTTGTATAGAGAAACTATTGGTTAGTCAAGTATG
GAAATGATCAAGACTAACAAATGCCGTTCTCCAAGGGCTATAAGTTTTGTTGTCATCAGTACGAGCAAAAGG
TTGATCAATAGCAATGGCTCATCTCCTGTTCTTGCCCATCCTGCAACTCTTGCTACTATACTGCACGAAATCTGC
CCAAGCTCAGCTAAACATCAGTATAGGCTCCTCTTTGACACCCCAGGGGGTAAACAACTCATGGATCTCGCC
CTCTGCTGATTTTGCATTCGGTTTTCGGGCGGTAGATGGAAACTCCTCCTCTTACCTGCTCGCCGTCTGGTTC
AACAAGATCGCTGACAAGACCGTCGTCTGGTACGCAAGGACTAGCTCCAATGGGAAAGATGATACAATACC
GGTACAAGTCCAATCTGGCTCTGTCCTCAAGCTCGCTGATGGAGCACTCTCTCTTCGCGATCCATCTGGCAAT
GAGGTATGGAATCCACAAGTCACTGATGTGGGCTATGCTAGAATGCTCGACACCGGGAATTTCAGGCTTTTA
GGCACCGATGGTGCAACAAAGTGGGAGTCCTTTGGTGACCCCTCTGATACCATCCTACCCACACAAGTGCTT
TCACTGGGGACGGCACTCCACAGCCGTCTCCTCGCCACAGACTATTCCAATGGCCGATTTCAACTAAAAGTT
CAACGAGATGGTAATCTTGTTATGTATCCAGATGCTGTACCTTCTGGATACTTATACGATCCATATTGGGCTAGT
AACACAGTGGACAATGGCTCACAGCTAGTGTTCAATGAAACTGGAAGGATATACTTCACTATAATTAATGGTT
CACAGGTAAATATCACTTCTGCAGGGGTGGATTCTATGGGTGATTTCTTCCATCGTGCTACCCTTGACACAGA
TGGTGTGTTCCGGCAATATGTTTACCCAAAAAACATACATGCCAGGCCCTTATGGCCAGAGCAATGGACTGC
AGTCGATGTACTTCCAGAAAACATCTGCCAGTCAATACAAACAATGGTGGGTAGTGGAGCATGCGGCTTTAA
CAGTTACTGCACCATTGATGGCACCAAGAACACGACAAGTTGTTTATGCCCACAGAATTATAAGTTCATCGAT
GATAAAAGGAAATACAAAGGCTGCAGGCCAGATTTTGAGCCACAAAACTGTGATCTGGATGAGACGACAG
CAATGTTGCAGTATGACATGGCACCAATCGATCGCGTTGATTGGCCTCTATCTGACTATGAGCAATACAATCCC
ATAGATCAGACCGAATGCCGAAGGCTGTGTGTGATTGATTGTTTCTGTGCCGTAGCTGTGTTCGATAAAGCTT
CAAGCACTTGTTGGAAGAAAAGGTTCCCTTTGTCGAACGGTAAAATGGATGTCAATGTACCGAGGACAGTT
CTTATTAAGGTGCCTAGGAGCACCAATTCACCATCCGTGTTCAGCAGCGGCTCAAGCAAATGGAAGGAGGA
CAAAAAGTATTGGATTCTTGGGAGTTCATTACTTTTTGGAAGCTCTGTATTGGTGAACTTTCTCCTAATCTCTG
TTATGCTTTTTGGTACTTATTGTAGTATCACCTCGAGGAAGAAAATCCAGTTATCGCAACCGTCCAATAAATCT
GGATTACCTCCAAAGATTTTCACTTACAGCGAGCTGGAAAAGGCAACCGGTGGTTTCCAAGAAGTACTTGG
CACAGGAGCCTCTGGTGTTGTCTACAAAGGACAGCTGCAAGATGAGTTTGGGACTAACATCGCGGTCAAGA
AAATCGAAAAGCTTCAGCAGGAAGCACAAAAGGAGTTCTTGGTGGAAGTCCAAACCATTGGGCAGACATT
TCACAGGAACTTAGTTAGACTGCTTGGTTTTTGCAACGAGGGAACTGAAAGGCTGTTAGTGTATGAGTTCAT
GAGCAATGGATCACTCAATACATTCCTCTTCAGCGATACCCATCCACATTGGAGCCTCCGTGTTCAAGTCGCA
CTTGGGGTGGCACGAGGACTGCTCTACCTACATGAGGAGTGTAATAAACAAATCATCCACTGCGATATGAAA
CCACAGAATATCCTTCTCGATGATAATTTTGCAGCAAAGATTTCAGATTTTGGCCTAGCAAAGCTTCTTCCGG

TGAATCAGACACAAACAAACACCGGCATTCGGGGTACTCGAGGATATGTTGCACCTGAGTGGTTCAAGAAC
ATAGGGATTACTTCTAAGGTCGACGTTTATAGCTTTGGGGTGATTTTGCTTGAGCTTGTGTGCTGCAGGAAG
AATGTGGAATTAGAAGTCCTAGATGAAGAGCAGACAATACTAACTTATTGGGCAAATGATTGCTACAAGTGT
GGGAGGATTGACTTGCTGGTAGCGGGCGATGACGAGGCAATCTTCAACATAAAAAAGGTGGAGCGCTTCG
TTGCTGTGGCACTGTGGTGCCTCCAGGAGGAGCCATCGATGCGGCCAACCATGCTTAAGGTGACACAAATG
CTTGATGGAGCAGTACAAATACCAACGCCTCCTGATCCTTCCTCCTATATCAGTTCACTTGCATGAAAACAGTC
TTCCTGTTAATAAATAGATTGTAGTTTGAAGCAAGAGCATGCTTTAGACTGATTCCTCAATAAGGCATTGTGTT
CGGGCACTGATGCTTACCTACAATAAGTAGCTTACATATCTAGAAAGATGCAGATAGATGCATCACTAGACCC

>195B-12600 (*bph3*)
TCAATCTTAGAAAAAGTATGTGGCTATCATGGTCTTTCTTAGAATGGAGGATGCAAGGGATGTTGTATGTATAT
GCAAATTTCAGTTCACAAAATCCAGGTGGTATATAGAAGGCCAGCTAAGACAATGACCATTTACCTACAGATA
CTATTCCTCGTGACATTTTTACTTAGAAATAACTTGTAAGGTGGGCAGGATCTCAAGTTGACTTGACCTTACA
AGTTCAAAAAAAAATAACTTGTAAGGTCAAGTCAACTCTTCGGTGTGCAATAAAAAGTAGGAAATGGAAGC
AATAATCAGAACAAGCAATATGGCACCTCCACTCTTCTTGCTCTCCCTCCAATTGTTGGTGCTACTGTCCTCCC
CATCTGCTCAAGCTCAGAACATCAGCTTGGGCACATCATTAACAACCCAAGGGCCAAGCAACGCATGGCTCT
CGCCATCAGGCGACTTCGCGTTTGGATTCCGGCCCATCGATGGCAACTCCTCCTTCTACCTCCTCGCCATCTG
GTTCAACAAGATCAGCGACAAGACGGCAACATGGTATGCCAAGACCAGTGAGCAAGAACCACAGCCGATA
CAAGTGCCATCTGGCTCCATCCTCCAGTTTACCTCAACTGGTGTACTTTCTCTCCGAGATCCCACCAATAGAG
AGGTATGGAATCCAGGAGCAACTGGTGCACCCTATGCCAGCATGCTCGACACCGGGAATTTTGTGATTGCTG
CTGCAGGAGGCTCTACTATCAGCTGGGAAACTTTCAAGAACCCAACAGACACCATCCTGGTCACACAAGCG
CTATCCCCTGGAATGAAGCTCCGCAGCCGTCTCCTTACCACAGATTACTCCAATGGCAGGTTTCTTCTTAACAT
GGAAACTCAACGAGCTGCACTTTATACCATGGCAGTTCCATCTGGAAACCTTTATGACCCATATTGGTCCACA
CCCATAGATGAGAATGTCACAAATCAGGTCACCAATCTGGTGTTCAATACTACCGGTAGGATATACGTGAGCA
TGAAGAATGGAACACAATTCAACATGACATCTGGGGTGATCCGCTCCATGGAGGACTACTACCATCGTGCTA
CGCTTGACCCAGATGGTGTGTTCAGGCAATATGTGTACCCGAAGAAGCCCAGCAGCATGAGTCAGGCATGG
ACAGCAGTGAGCATCCAACCTGAAAACATCTGCAATGCTCAGACAAAAGTAGGCAGTGGCACCTGTGGATT
CAATAGTTACTGCATGTTTGATGGCAGCAACAACCAGACAAGCTGTGTGTGCCCGGAGCAGTACTCGTTCTT
TGATGAGGTGAGGAAGTACAGAGGCTGCAGACCGGACTTCGAGCTACAAAGCTGTGATTTGGATGAGGCA
GCTTCCATGGCACAGTATGAGTTTAACTTGGTTAATAATGTGGATTGGCCTCAGGCTGACTATGAGTGGTACA
CTCCCATAGACATGGATGAATGTCGACGGCTCTGCCTGATTGATTGCTTCTGTGCTGTTGCTGTATTCCATGAA
AACACCTGCTGGAAGAAGAAGCTCCCTCTATCAAACGGGATCATGGGGAGTGGAGTGCAGAGAACAGTTC
TTATCAAGGTGCCAAAGAGCAACAGTTCGCAGCCAGAGCTCAGAAAGTCTAGAAAATGGAAGAGTGACAA
GAAGCTCTGGATCCTAGGAAGTTCGTTGCTTCTCGGAGGCTCTGTGATAGCGAATTTTGCCTTGAGTTCTGT
TCTTCTTTTCGGTACTTACTGTACCATCACCAGAAAGGATGTCCAGCCATTGCAACCATCACGAGACCCAGGA
TTACCCCTCAAAGCTTTCAGTTATGCAGAGCTTGAGAAGGCAACGGACGGATTCAAGGAGGTGTTGGGCAC
AGGTGCATCTGGTaTTGTGTACaAAGGCCAGCTGCAAGATGAATTAGGAACTTACATTGCTGTCAAGAAAAT
TGATAAGATTCAGCATGAGACTGAAAAGGAGTTTGCTATGGAGGTCCAAACCATTGGACGGACGTACCATA
AGAACCTGGTCCGGATGCTAGGATTCTGCAATGAAGGAACTGAGAGACTGTTGGTGTATGAATTCATGGTTA
ATGGATCGCTCAACAGATTCCTGTTTAGTGGTGTCAGGCCTCTGTGGAGTCTTCGAGTTCAACTTGCTCTCG
GTGTCGCAAGGGGGCTGCTATACTTACATGAGGAATGCAGCACACAGATCATCCACTGTGACATAAAGCCCC
AAAATATCCTTCTTGATGATAATTTCATAGCAAAGATTTCAGACTTTGGCTTAGCGAAACTACTCCGAACCAAC
CAAACACAGACATATACAGGTATCCGTGGTACCCGTGGATATGTTGCCCCTGAGTGGTTCAAGAACGTCGGG
ATCACTGCTAAGGTGGATGTGTACAGCTTTGGGGTCATTCTTCTGGAACTCATCTGTTGCCGGCAAAATGTG

GAAATGGAGGCTGCAGAAGAAGAGCAAAGTATTCTTACTTACTGGGCAAATGACTGTTATAGGTGTGGCAG
GGTCGACCTGCTGGTAGATGGTGATGATGAGGCGAAGTTAAACATCAAGAAGGTTGAAAGGTTTGTAGCA
GTGGCATTGTGGTGTCTCCAGGAGGAGCCAACTATGCGACCCAGCATCCTGAAGGTAACTCAAATGCTTGAT
GGAGCGGATGCAATCCCAACTCCTCCAGATTCATCTTCTGTGGTCAACTCGTTTCCATAATGTACTGCCTAGAT
ATTGAATAATTAATGCATGTATAAACTAACATCAACATTTATGGTGTGGAAAGTTATATAATTAACAACAATTAA
CTTTGTTCTCAGGCGCATATTTAGCCCTGAACATGTCATAGAATTTAAAACCAAGCATTATATAATAAATATAGC

>195B-*Bph2*
ATGGAGGCCACGGCGGTGAGCATTGGCAGGTCCGTGCTGAAGGGAGCGCTTGGCTTCGCCAAATCCACCT
TGGTGGAGGAGGTTTCCCTGCAGCTCGGCGTCCAGCGTGACCAGGCGTTCATCAGGGACGAGCTGGAGAT
GATGAACTCCTTCCTGATGGCCGCCAATGATGAGAAAGATGACAACAAGGTGGTAAGGACCTGGGTGAAG
CAGGTCCGCGACGTGGCCTACGACGTCGAGGACTGCCTCCAGGACTTCGCCGTCCGCTTGGGGAGGAAGA
GTTCATCCTGGTGGCTCAGCCCTCACACGCTGTGGGAGCGGCGCCGCATCGCCAAGCAGATGGAGGAGCT
GAGGGGCAAGGTTGAGGATGTGAGCCAGAGGAACATGCGTTACCAACTCATCAAGGGCTCCAAGCCTACC
GTAGCTACCAATGTCGCACCCAGCAGCACTGCCCGTGCGACCATGTCTGGCGCGCATGAAGAACGATGGCA
GCATGAGAAGGCAATAGATCATCTGGTTCGGCTGGTCAAAACCAAAGTCGATGAACGTAGAGTGATCGCGG
TGTGGGGAACAAGTGGTGATATCAGGGAAATGTCCATCGTTGGAGGGGCCTATGATCATCTCAAGAGAAGC
AACAAGTTTGAGTGCTGTGCCTGGGTTAATTTGATGCATCCTCTGAACCCAACAAAGCTCCTGCAAACCATT
GTTAGGCAATTCTATGTAAGATCTCTTCAGGAGGCTGGCAAAGCAACTCCGTCGTGTCAAATTCTGAGTAGC
ATGCTGATAAAGGAAGATCATTTGAACGATGAGTTCAATGAATATTTGAGTGACAAGTGCTACCTCGTTATGC
TTAATGACCTATCAACCGCTGAAGAATGGAAGCAAATAAAAATGCTCTTCCCAGACAACAAGAAAGGGAGC
CGAATCATAGTGTTCACACAACATGTTGAAGTTGCAAGCTTTTGTGCTAGGACCGAGGAGGTGGCACCCGA
GCAAATGCAGCTGTTTGCTGATCAGACTCTTTATGCTTTTCGCTGTAAGGTACTCCATCCGTTCCAAAATGATC
ATCATATAGTTTTTTTTAGGTTATTTCTAAATAATTATCATATTTATATTCATTCATTATGTATATTCGTTATTTGTTC
ATTGGAGTAAATGGATATTGATGCATGTATCAGTGCACACAAGTATTTATAACCCACATGCAATATCTTGATTTG
CTATTGGCTAGGAAATAGTGGAATGGTGCATGCATCGAGTTTGTTGCTAGAGTAAATATAGTATGAGAGAGTT
ATTAGCTTTTCTTGATATTGGTGTACCTATGAAATATGTAGATCAATTTAGAATGGAGGAAGTAATACTAAAAA
AAAATCCTCAAACATGCACTGAAAAAGAAATATTCTATATATCATCATCCGTTGAAAGCCTACTCTTTTCATTCA
ACTTGTCTGCTAATTAAATCAATGTTTTATCTATGTCATTATGTACAAAAGAAACTAACACTGATTAATAGTACG
ACATAGCTCTGTCTCTTTCATCTCTAAGAACATCTCCAGCAAACATATATCATATTCGCTATAGTTATAATTCAAC
AATTCTCTTTTAAAAAAATAACAGCTCCAATAAATCGCCCTACTCAATTTTAGGTACTCCCCATAGTGAGTATAAT
CGACCCCAAATTTTGGGTGCCCCTGTCCCACTCCCTATGCCAGCCAAGACTTGTTGATCGCCAGGGGCGGAA
CCACCCATTAGGTAGGGTTGGGCGGCCAGCCCAGCTATGGCCCGCGCCGCCCCAACTCCCCATATCCTTCTT
GGGCCGCCTACCACCACATCCAATTCACAATTGGCCCATAAAAAAGCAGCCCAAGTGTCCAATTCAGATTTT
CTGGGAAATCAAGAGTTAAACTCATGCCCGCGATGCCTCGGTACCTAGCGCCATCATCTCCGCATGCTGCGTC
TCGCTACCCACACGCGACACCTCGCTACCCGCACGTGACGCGTCGCTTCGCTACCTGAGCGTGCCTAGGCTA
GCCGCCGCCCGCTGGCGCCTAGCGACGGCCGACACCATTCGCTCCTCCTGCTCGGCGCCACACGCGCCAAC
TCGACGACATTACCTCGCCATCGCCCCGTCCCTACCGAGTTCCAGCCATTCCCGCGCCAACGCCGCCGCCCG
GTCATCGGCAGCACGCAGCACCGCAGGCCGCAACAGCCAGTAGCCCAGTAGCTTGCGGCGGCTTTAGCATC
GGCCATCGGGCATCCTTGAGAATGCTCACCCCCATCACCTACTATAGTTAGGTGCCTAACGTTTTTCTCTCTCT
CCCTTCTAAGCAAAATAATAATCATCTTATTCGGGGTATGTCTTCTCATATATTTATTTTTTTAAAGATATCTATCA
ACCATGCTTTATGAATTGACTCATATTTGCAAATTGATACGTTTACCAATATCTTTTTATGTATGATGAATTATTTT
GGCGCAAAAATTATTACTTTATTATAATATCGTTTTTGTTTGCTTTAATTTATTGATTATACTATATGTTTATGTGA
TTACAACATAAATATAGTATTTGTTGCATTTAGTTGTTCTTATTGACACGAGCATCTTCGCCCTATCTTTAATTTA

ATCCTGCGTCCGCCACTGTTGATCGCTATCATTTTCACTGCATCCTTCACCTTCCTCTCCTCCCCACATCCCTCA
TCTTGCTTGGCCTTCGCCTCCTGCGACCACCACTTAGCTGGCCATTGCTCTTTGCCCCATCAAGCTCCAACCT
CTGTTGAAAGCATATTTTATTTTGCATGTTGGAACGGCAACTCCATCTTAAACTTACTGCTTAAAAGCCAACA
ATTTGTAAGCTATATAAGCCAAAATGCCGGCTTATAGGCATACTATACTAAGACTGTGATTAATTTGCTTGTATT
TAGTATTATGCAATACGGTAATACTTTGAGCTCAACTTTAACTTATACTACATAACACATTTTTAGAGGATTTTG
AAGTTCAAAAATGTGTAGATAGGTGCATTTGCACTTATCTGAAAATTCCCAAGGATACTTGATTATTATGTACT
ATAATCAGAATCCCACCAAATTTACAGATTGATAAATCTTTTAATTGATTGGGCAAGTAATATATGGAGTAACA
CTTATGGGTCGGGAGGCATTTAAAAATTAAGTAGTTCAATTGAGATTGTCTTTTCACTAACGAATGGTAACCT
ATTTATACATCTACAAGAGATTTGTCTTGGGAAACAACTAAGTATTATCATAGGAAAGTAGACAATTAGACATC
ATCATCATCTAATATTCTAAATAATAACTGTTCTCAAGAGGTTGGCTACATTTTTTTGCTGTTGTTTCATTGAAA
AGTTCTTGCAACCTTTTTTCCATTAAGGGCATGTACGATTCCTTTTTAACTCTCGTCTCGTCTTAAAATATAATG
GTGCACACCTATCATGCGTGTTTAAGAAAATAATATTGTTGATAATATTTTTTTTTTGTGTGTGCAGGGTGCTA
AAGATGGAGTAGATTCAATGGAGGACTCATCTAACTTAAACGAAGACACTACATACAACGCTGTAGAAGGA
AAGAGCCTCCCTCGCACATATTCAATGGTAACTGCTTTCAAGGAATCTGAGATCGTTGGGCGAGTTGATGAA
ATAAAGGAGATTATTGAACTGATTTCAAAAGGTAGCCAACAGCTTGAGAAGATCTCAGTGTGGGGAATGGG
TGGTATTGGGAAAACCACTCTAATTCAAAATGTCTACCGAAGCGAAAAGGTTAAGAAGATGTTTGATAAGCA
TGCATGTGTCACGATCATGCGCCCGTTCAATCTTAATGATCTTCTTATGAGCTTAGTTAGGCAACTAGAAGATT
CAAAAACTTCTGGAGAAAAGGAGTTGGCTAGCATTTTAGAAGGAAAGAAATACTTGATTGTTCTTGATGAT
GTATTATCCACAACAGAATGGAATGCTATAGAATCATATTTCCCAGCAATGGAAACAGGAAGCCGGATCATAA
TCACCACAAGGCATGAAAGTATTGCTAAGCATTGTTCAGGGGATCAACAAGGAAAAATATATCAACTCAATC
GTCTAGGAGACAGCGATGCAAAGAACCTTTTTGCAAAAAAGGTAACCTAA

Figure S4. The *bph3* and *bph2* genome sequences of the maintainer line 195B. The *bph3* gene cluster contains four gene segments, 195B-12540, 195B-12560,195B-12580 and 195B-12600.
