# Supplementary material for: Bph32, a novel gene encoding an unknown SCR domain-containing protein, confers resistance against the brown planthopper in rice
Source: Sci Rep. 2016 Nov 23;6:37645. doi: 10.1038/srep37645 (PMC5120289; doi:10.1038/srep37645)
Supplement: Supplementary Table S1 [file srep37645-s5.pdf]

Bph32， a novel gene encoding an unknown SCR domain-containing protein confers resistance against the brown planthopper in rice

Juansheng Ren[1]*, Fangyuan Gao[1]*, Xianting Wu[1]*, Xianjun Lu[1], Lihua Zeng[3], Jianqun Lv[1], Xiangwen Su[1], Hong Luo[2], Guangjun Ren[1]**

[1]Crop Research Institute, Sichuan Academy of Agricultural Sciences, Chengdu, 610066, P.R. China

[2]Department of Genetics and Biochemistry, Clemson University,110 Biosystems Research Complex, Clemson, SC 29634-0318,USA

[3]Sichuan Normal University, Chengdu, 610066, P.R. China

*These authors contributed equally to the work.

**Corresponding author e-mail: guangjun61@sina.com

Table S1. Prediction of potential genes and potential protein domains by FGENESH 2.6 and SMART, respectively.

Predicted gene		MSU LOCUS ID	MSU Rice Genome Annotation	Predicted domain (E-value<0.1)
In Nipponbare	In 9311(gene or allele)			
FGENESH 1	FGENESH 1	LOC_Os06g03220.1	expressed protein	transmembrane region;low complexity
	FGENESH 2			Pfam:DUF390
	FGENESH 3			eleven low complexities; Retrotrans_gag; RVT_1;RVT_3; rve

	FGENESH 4			two low complexities; 4MH8|A
	FGENESH 5			signal peptide; rve
	FGENESH 6			
FGENESH 2	FGENESH 7	LOC_Os06g03240.1	expressed protein	See Table S
FGENESH 3	FGENESH 8	LOC_Os06g03250.1	retrotransposon protein, putative, Ty3-gypsy subclass, expressed	
FGENESH 4		LOC_Os06g03260.1	retrotransposon protein, putative, unclassified, expressed	
FGENESH 5		LOC_Os06g03270.1	retrotransposon protein, putative, unclassified, expressed	
FGENESH 6	FGENESH 9	LOC_Os06g03280.1	retrotransposon protein, putative, Ty3-gypsy subclass	
FGENESH 7	FGENESH 10	LOC_Os06g03290.1	conserved hypothetical protein	

FGENESH 8	FGENESH 11	LOC_Os06g03300.1	retrotransposon protein, putative, unclassified	
FGENESH 9	FGENESH 12	LOC_Os06g03310.1	retrotransposon protein, putative, unclassified, expressed	
FGENESH 10	FGENESH 13	LOC_Os06g03320.1	retrotransposon protein, putative, unclassified, expressed	
FGENESH 11	FGENESH 14	LOC_Os06g03330.1	hypothetical protein	
FGENESH 12	FGENESH 15	LOC_Os06g03340.1	retrotransposon protein, putative, Ty1-copia subclass, expressed	
FGENESH 13		LOC_Os06g03360.1	retrotransposon protein, putative, Ty1-copia subclass	
FGENESH 14	FGENESH 16	LOC_Os06g03370.1	retrotransposon protein, putative, Ty3-gypsy subclass, expressed	
FGENESH 15	FGENESH 17	LOC_Os06g03380.1	expressed protein	
FGENESH 16	FGENESH 18	LOC_Os06g03390.1	expressed protein	

FGENESH 17		LOC_Os06g03400.1	retrotransposon protein, putative, Ty3-gypsy subclass	
FGENESH 18		LOC_Os06g03410.1	retrotransposon protein, putative, Ty3-gypsy subclass, expressed	
FGENESH 19		LOC_Os06g03420.1	retrotransposon protein, putative, Ty3-gypsy subclass, expressed	
FGENESH 20		LOC_Os06g03430.1	retrotransposon protein, putative, Ty3-gypsy subclass, expressed	
FGENESH 21		LOC_Os06g03440.1	retrotransposon protein, putative, Ty3-gypsy subclass	
FGENESH 22		LOC_Os06g03450.1	retrotransposon protein, putative, Ty3-gypsy subclass	
FGENESH 23		LOC_Os06g03460.1	retrotransposon protein, putative, Ty3-gypsy subclass, expressed	

FGENESH 24		LOC_Os06g03474.1	retrotransposon protein, putative, Ty3-gypsy subclass, expressed	
FGENESH 25				coiled coil; low complexity; d1tf4a1
FGENESH 26	FGENESH 19			low complexity
FGENESH 27	FGENESH 20	LOC_Os06g03500.1	NBS-LRR disease resistance protein, putative, expressed	
FGENESH 28	FGENESH 21			low complexity
FGENESH 29	FGENESH 22	LOC_Os06g03520.1	DUF581 domain containing protein, [illegible]	zf-FLZ;low complexity
FGENESH 30	FGENESH 23	LOC_Os06g03530.1	pentatricopeptide, putative, expressed	six pentatricopeptide repeats (PPRs)
FGENESH 31	FGENESH 24	LOC_Os06g03540.1	oligopeptide transporter, putative, expressed	two low complexities;OPT
FGENESH 32	FGENESH 25	LOC_Os06g03560.1	oligopeptide transporter, putative, expressed	glutathione transporter

FGENESH 33	FGENESH 26	LOC_Os06g03570.2	pentatricopeptide, putative, expressed	pentatricopeptide (PPR) repeat-containing protein-like
FGENESH 34	FGENESH 27	LOC_Os06g03580.1	zinc RING finger protein, putative, expressed	
FGENESH 35	FGENESH 28	LOC_Os06g03590.1	retrotransposon protein, putative, unclassified, expressed	
FGENESH 36	FGENESH 29	LOC_Os06g03600.2	transcriptional corepressor SEUSS, putative, expressed	
FGENESH 37	FGENESH 30	LOC_Os06g03610.1	TKL_IRAK_CrRLK1L-1.13 - The CrRLK1L-1 subfamily has homology to the CrRLK1L homolog, expressed	
FGENESH 38		LOC_Os06g03620.1	expressed protein	three low complexities
FGENESH 39	FGENESH 31	LOC_Os06g03630.1	expressed protein	
