# Supplementary material for: Bph32, a novel gene encoding an unknown SCR domain-containing protein, confers resistance against the brown planthopper in rice
Source: Sci Rep. 2016 Nov 23;6:37645. doi: 10.1038/srep37645 (PMC5120289; doi:10.1038/srep37645)
Supplement: Supplementary Table S2 [file srep37645-s6.pdf]

# *Bph32*， a novel gene encoding an unknown SCR domain-containing protein confers resistance against the brown planthopper in rice

Juansheng Ren[1]*, Fangyuan Gao[1]*, Xianting Wu[1]*, Xianjun Lu[1], Lihua Zeng[3], Jianqun Lv[1], Xiangwen Su[1], Hong Luo[2], Guangjun Ren[1]**
[1]Crop Research Institute, Sichuan Academy of Agricultural Sciences, Chengdu, 610066, P.R. China
[2]Department of Genetics and Biochemistry, Clemson University,110 Biosystems Research Complex, Clemson, SC 29634-0318,USA
[3]Sichuan Normal University, Chengdu, 610066, P.R. China
*These authors contributed equally to the work.
**Corresponding author e-mail: guangjun61@sina.com

**Table S2a. Prediction of domains (or repeats, motifs and features)in TN1 and Ptb33 by SMART**

| Gene from variety | Confidently predicted domains | STart | End | E-value |
|---|---|---|---|---|
| TN1 | Signal peptide | 1 | 29 | N/A |
| | Low complexity | 120 | 133 | N/A |
| | SCOP d1g6xa_ | 166 | 181 | 0.026 |
| | Transmembrane | 10 | 32 | N/A |
| Ptb33 | Signal peptide | 1 | 29 | N/A |
| | Low complexity | 120 | 133 | N/A |
| | SCOP d1g6xa_ | 166 | 181 | 0.026 |
| | Transmembrane | 10 | 32 | N/A |

Table S2b. Prediction of Protein-Protein and Protein-Polynucelotide Binding Sites in TN1 and Ptb33 by PredictProtein

| Gene from variety | Binding Sites | Length | Type | ID | Evidence |
|---|---|---|---|---|---|

| | | | | | |
|---|---|---|---|---|---|
| TN1 | 1 | 1 | Protein [illegible] | SO:00 00410 | ISIS |
| | 40 | 1 | Protein binding region | | |
| | 71 | 1 | Protein binding region | | |
| | 76-78 | 3 | Protein binding | | |
| | 87-93 | 7 | Protein binding | | |
| | 120-124 | 5 | Protein binding | | |
| | 135 | 1 | Protein binding | | |
| | 155-157 | 3 | Protein binding | | |
| | 173 | 1 | Protein binding | | |
| | 180 | 1 | Protein binding | | |
| | 190 | 1 | Protein binding | | |
| | 194 | 1 | Protein binding | | |
| | 42 | 1 | Protein binding region | SO:00 00410 | ISIS |
| | 44 | 1 | Protein binding region | | |
| | 55-56 | 2 | Polynucelotide-binding region | SO:00 01429 | SomeN[illegible] |

| | | | | | |
|---|---|---|---|---|---|
| Ptb33 | 58 | 1 | Polynucelotide-binding region | SO:0001430 | A |
| | 69 | 1 | Protein binding region | SO:0000410 | ISIS |
| | 135-136 | 2 | Protein binding | | |
| | 156 | 1 | Protein binding region | | |
| | 157-158 | 2 | Polynucelotide-binding region | SO:0001430 | SomeNA |
| | 184 | 1 | Protein binding | SO:0000410 | ISIS |
| | 194 | 1 | Protein binding region | | |
