# Supplementary material for: Bph32, a novel gene encoding an unknown SCR domain-containing protein, confers resistance against the brown planthopper in rice
Source: Sci Rep. 2016 Nov 23;6:37645. doi: 10.1038/srep37645 (PMC5120289; doi:10.1038/srep37645)
Supplement: Supplementary Table S3 [file srep37645-s7.pdf]

Bph32, a novel gene encoding an unknown SCR domain-containing protein confers resistance against the brown planthopper in rice

Juansheng Ren[1]*, Fangyuan Gao[1]*, Xianting Wu[1]*, Xianjun Lu[1], Lihua Zeng[3], Jianqun Lv[1], Xiangwen Su[1], Hong Luo[2], and Guangjun Ren[1]**

[1]Crop Research Institute, Sichuan Academy of Agricultural Sciences, Chengdu, 610066, P.R. China

[2]Department of Genetics and Biochemistry, Clemson University,110 Biosystems Research Complex, Clemson, SC 29634-0318,USA

[3]Sichuan Normal University, Chengdu, 610066, P.R. China

*These authors contributed equally to the work.

**Corresponding author e-mail: guangjun61@sina.com

Table S3. Respones of *Bph32* transgenic $T_{2:3}$ plants, wild-type variety and other main marterials to BPH infestation in this paper

Trait / vartiety	Scale of plant damage*	Honeydew area** (mm^2)	Nymph survival number[#]
***Bph32* transgenic $T_{2:3}$ plant(N65-7-1-1-8)**	3.6±1.2	22.44±13.15	1.5±0.5
**wild-type variety (kasalath)**	8.7±0.6	197.91±45.65	14.8±1.6
**Ptb33**	0.7±0.6	11.88±12.33	
**Taichung Native (TN1)**	9.0±0	210.60±47.61	
**195B**	3.6±1.2		
**195A/Chenghui3203**	3.0±0		
**163B**	9.0±0		
**Replication**	3	18	10

Note: All data are given as mean±SD. *, the scale was recorded when the TN1 plants were died at seedling stage. **, 24 hours after BPH infestation, the filter papers were collected; total area of blue-green spots was measured using Image-Pro Plus 6 program. #,the number of BPH on each plant was recorded at 13 day post-infestation.
