# Supplementary material for: Bph32, a novel gene encoding an unknown SCR domain-containing protein, confers resistance against the brown planthopper in rice
Source: Sci Rep. 2016 Nov 23;6:37645. doi: 10.1038/srep37645 (PMC5120289; doi:10.1038/srep37645)
Supplement: Supplementary Table S4 [file srep37645-s8.pdf]

Bph32, a novel gene encoding an unknown SCR domain-containing protein confers resistance against the brown planthopper in rice

Juansheng Ren[1]*, Fangyuan Gao[1]*, Xianting Wu[1]*, Xianjun Lu1, Lihua Zeng[3], Jianqun Lv[1], Xiangwen Su[1], Hong Luo[2], and Guangjun Ren[1]**

[1]Crop Research Institute, Sichuan Academy of Agricultural Sciences, Chengdu, 610066, P.R. China

[2]Department of Genetics and Biochemistry, Clemson University,110 Biosystems Research Complex, Clemson, SC 29634-0318,USA

[3]Sichuan Normal University, Chengdu, 610066, P.R. China

*These authors contributed equally to the work.

**Corresponding author e-mail: guangjun61@sina.com

Table S4. Plant material used in this paper.

**Accession or variety***	**origin country or region**	**taxon**	**BPH resistance gene****	**Scale**
121216	China	*Oryza sativa*	*Bph32*	3.6
195B	China	*Oryza sativa*	*Bph32*	3.6
BC-10-46	Philippines	*Oryza sativa*	*Bph32*	2.3
DARMALI	Nepal	*Oryza sativa*	*Bph32*	4
GPNO-25912	El Salvador	*Oryza glaberrima*	*Bph32*	4
IR54751-1-2-44-15-2-3-B	Philippines	*Oryza sativa*	*Bph32*	1
IR60	Philippines	*Oryza sativa*	*Bph32*	4.3

IR64-D-518-6-106	Philippines	*Oryza sativa*	*Bph32*	3
IR64-D-518-6-	Philippines	*Oryza sativa*	*Bph32*	3
IR70	Philippines	*Oryza sativa*	*Bph32*	4.3
IR78222-20-7-148-2-B	Philippines	*Oryza sativa*	*Bph32*	3
IR78525-150	Philippines	*Oryza sativa*	*Bph32*	2.3
IR80314-4-B-1-3-B	Philippines	*Oryza sativa*	*Bph32*	3
IR80340-23-B-12-6-B	Philippines	*Oryza sativa*	*Bph32*	3
Oryza	Bolivia	*Oryza latifolia*	*Bph32*	
Ptb33	India	*Oryza sativa*	*Bph32, Bph3*[p], *BPH26*	0.6
SHZ-2		*Oryza sativa*	*Bph32*	2.3
Tin-Tin95	Myanmar	*Oryza sativa*	*Bph32*	1.3
UA-1012	Niger	*Oryza sativa*	*Bph32*	3
Yuanhong-Wildrice[#]	China	ryza nivara	*Bph32*	
Zengxiang	Myanmar	*Oryza sativa*	*Bph32*	1
IR65482-4-136-2-2-B	Philippines	*Oryza sativa*	*Bph10*[84], *bph32*[389B]	3
Bombon	Spain	*Oryza sativa*	*bph32*[389B]	9
WC2811	Micronesia	*Oryza sativa*	*bph32*[389B]	9
BERLIN	Costa Rica	*Oryza sativa*	*bph32*[389B]	9
Padi Pohon Batu	Malaysia	*Oryza sativa*	*bph32*[389B]	9
Italica Carolina	Poland	*Oryza sativa*	*bph32*[389B]	9
KRASNODAR SKIJ 3352	Russian Federation	*Oryza sativa*	*bph32*[389B]	9
Hi Muke	Kazakhstan	*Oryza sativa*	*bph32*[389B]	9
Chuanxiang29	China	*Oryza sativa*	*bph32*[389B]	9
JIN23B	China	*Oryza sativa*	*bph32*[389B]	9
WC-3532	Peru	*Oryza sativa*	*bph32*[389B]	9
LUSITANO	Portugal	*Oryza sativa*	*bph32*[389B]	9

389B	China	*Oryza sativa*	$bph32^{389B}$	9
SORNAVARI	Mali	*Oryza sativa*	$bph32^{389B}$	9
Rathu Heenati	Sri Lankan	*Oryza sativa*	*Bph3*， $bph32^{389B}$	1
Kasalath (Ka)	India	*Oryza sativa*	$bph32^{ka}$	9
Stg 9544-32	United States	*Oryza sativa*	$bph32^{ka}$	9
British-Honduras-Creole	Belize	*Oryza sativa*	$bph32^{ka}$	9
Bombilla	Spain	*Oryza sativa*	$bph32^{ka}$	9
THAVALU	Sri Lanka	*Oryza sativa*	$bph32^{ka}$	9
Ardito	Italy	*Oryza sativa*	$bph32^{ka}$	9
Toga	India	*Oryza sativa*	$bph32^{ka}$	9
IR80310-12-B-1-3-B	Philippines	*Oryza sativa*	Unknown,$bph32^{ka}$	3
Red Khosha Cerma	Afghanistan	*Oryza sativa*	$bph32^{SK}$	9
Safut-Khosha	Afghanistan	*Oryza sativa*	$bph32^{SK}$	9
P-35	India	*Oryza sativa*	$bph32^{SK}$	9
WAB450-24-2-3-P33-HB	Cote D'Ivoire	*Oryza sativa*	$bph32^{p}$	9
IRRI-IRGC-101508	Philippines	*Oryza sativa*	$bph32^{TC}$	9
FIROOZ	Iran	*Oryza sativa*	$bph32^{TC}$	9
TCHAMPA	Iran	*Oryza sativa*	$bph32^{TC}$	9
Nam Dawk	Thailand	*Oryza sativa*	$bph32^{TC}$	9
Oryza rufipogon	India	*Oryza rufipogon*	$bph32^{Or}$	
Oryza glumipatula	Liberia	*Oryza glumipatula*	$bph32^{Or}$	
TOg-7147	Senegal	*Oryza glaberrima*	$bph32^{Or}$	9
Montakcl	Egypt	*Oryza sativa*	$bph32^{Or}$	9
B805D-MR-16-8-3	Indonesia	*Oryza sativa*	$bph32^{Or}$	9
Gasym-Hany	Azerbaijan	*Oryza sativa*	$bph32^{Or}$	9
Dara	Indonesia	*Oryza sativa*	$bph32^{Or}$	9

IR77186-122-2-2-3	Philippines	*Oryza sativa*	Unknown,*bph32*	3
IR77542-551--1-1-1-1-2	Philippines	*Oryza sativa*	Unknown,*bph32*	3
Y58S	China	*Oryza sativa*	*bph32*	9
TOg 6804	Nigeria	*Oryza glaberrima*	*bph32*	9
CG 14	Cote D'Ivoire	*Oryza glaberrima*	*bph32*	9
IR 58614-B-B-8-2	Philippines	*Oryza sativa*	*bph32*	9
E B Gopher	United States	*Oryza sativa*	*bph32*	9
Warrangal-Culture-1252	India	*Oryza sativa*	*bph32*	9
Sel. No. 388	Uruguay	*Oryza sativa*	*bph32*	9
Chin Chin	Panama	*Oryza sativa*	*bph32*	9
Eiko	France	*Oryza sativa*	*bph32*	9
NORIN-8	Japan	*Oryza sativa*	*bph32*	9
PI 298967-1	Australia	*Oryza sativa*	*bph32*	9
NSGC 5953	Myanmar	*Oryza sativa*	*bph32*	9
WAB462-10-3-1	Cote D'Ivoire	*Oryza sativa*	*bph32*	9
ARC-10633	India	*Oryza sativa*	*bph32*	9
HG-24	Burkina Faso	*Oryza sativa*	*bph32*	9
TOG-7102	Mali	*Oryza glaberrima*	*bph32*	9
TOg-7267	Cameroon	*Oryza glaberrima*	*bph32*	9
Romeno	Portugal	*Oryza sativa*	*bph32*	9
TOg-7161a	Senegal	*Oryza glaberrima*	*bph32*	9
HKG 98	Mali	*Oryza sativa*	*bph32*	9
Karabaschak	Bulgaria	*Oryza sativa*	*bph32*	9
TOg-7257	Chad	*Oryza sativa*	*bph32*	9
II-32B	China	*Oryza sativa*	*bph32*	9
CHENGHUI448	China	*Oryza sativa*	*bph32*	9
CHENGHUI3203	China	*Oryza sativa*	*bph32*	9
ASWINA-330	Bangladesh	*Oryza sativa*	*bph32*	9

Vary-Tarva-Osla	Portugal	*Oryza sativa*	*bph32*	9
Somewake	Japan	*Oryza sativa*	*bph32*	9
BHIM-DHAN	Nepal	*Oryza sativa*	*bph32*	9
MOROBEREKAN	Guinea	*Oryza sativa*	*bph32*	9
Won-Son-Zo-No.-11	Korea	*Oryza sativa*	*bph32*	9
IARI-6621	India	*Oryza sativa*	*bph32*	9
IR75870--8-1-2-B-6-1-1-B	Philippines	*Oryza sativa*	Unknown,*bph32*	3
Luxiang90B	China	*Oryza sativa*	*bph32*	9
Lemont	United States	*Oryza sativa*	*bph32*	9
TOg 7131	Senegal	*Oryza glaberrima*	*bph32*	9
Celiaj	Azerbaijan	*Oryza sativa*	*bph32*	9
CAROLINO 164	Chad	*Oryza sativa*	*bph32*	9
163B	China	*Oryza sativa*	*bph32*	9
Niwahutaw Mochi	Japan	*Oryza sativa*	*bph32*	9
Angkrang	Cambodia	*Oryza sativa*	*bph32*	9
CSORNUJ	Hungary	*Oryza sativa*	*bph32*	9
R 67	Senegal	*Oryza sativa*	*bph32*	9
N-2703	Nepal	*Oryza sativa*	*bph32*	9
WIR 3039	Tajikistan	*Oryza sativa*	*bph32*	9
K8C-263-3	Suriname	*Oryza sativa*	*bph32*	9
Chun 118-33	China	*Oryza sativa*	*bph32*	9
Jumli dhan	Nepal	*Oryza sativa*	*bph32*	9
INIAP 7	Ecuador	*Oryza sativa*	*bph32*	9
P 79	India	*Oryza sativa*	*bph32*	9
Chacareiro-Uruguay	Uruguay	*Oryza sativa*	*bph32*	9
Doble-Carolina	Uruguay	*Oryza sativa*	*bph32*	9
Hsin Hsing Pai Ku	Taiwan	*Oryza sativa*	*bph32*	9

Sapundali Local	India	*Oryza sativa*	*bph32*	9
TD 70	Thailand	*Oryza sativa*	*bph32*	9
IR 2061-214-2-3	Philippines	*Oryza sativa*	*bph32*	9
DJ-24	Bangladesh	*Oryza sativa*	*bph32*	9
TAINUNG 45	Taiwan	*Oryza sativa*	*bph32*	9
HB-6-2	Hungary	*Oryza sativa*	*bph32*	9
9311		*Oryza sativa*	*bph32*	9
Nipponbare	Japan	*Oryza sativa*	*bph32*	9
Taichung Native (TN1)	Taiwan	*Oryza sativa*	*bph32*	9

*Rice marterials were from International Rice Research Institute, Dale Bumpers National Rice Research Center, US Department of Agriculture-Agricultural Research Service (USDA-ARS) and Sichuan Academy of Agricultural Sciences.# Leaves were collected from Guangxi Academy of Agricultural Sciences. **Only major BPH resistance genes are listed.
