# Supplementary material for: Bph32, a novel gene encoding an unknown SCR domain-containing protein, confers resistance against the brown planthopper in rice
Source: Sci Rep. 2016 Nov 23;6:37645. doi: 10.1038/srep37645 (PMC5120289; doi:10.1038/srep37645)
Supplement: Supplementary Table S5 [file srep37645-s9.pdf]

Bph32，a novel gene encoding an unknown SCR domain-containing protein confersresistance against the brown planthopper in rice

Juansheng Ren[1]*, Fangyuan Gao[1]*, Xianting Wu[1]*, Xianjun Lu[1], Lihua Zeng[3], Jianqun Lv[1], Xiangwen Su[1], Hong Luo[2], Guangjun Ren[1]**

[1]Crop Research Institute, Sichuan Academy of Agricultural Sciences, Chengdu, 610066, P.R. China

[2]Department of Genetics and Biochemistry, Clemson University,110 Biosystems Research Complex, Clemson, SC 29634-0318,USA

[3]Sichuan Normal University, Chengdu, 610066, P.R. China

*These authors contributed equally to the work.

**Corresponding author e-mail: guangjun61@sina.com

Table S5. List of polymorphic markers among Ptb33, 195B, and 163B.

Marker	Chromosame	Physical distanc	Ptb33	195B	163B
RM1282	1	0.55	A	B	B
RM10054	1	1.06	A	B	B
RM7383	1	3.48	A	B	B
RM3235	1	7.51	A	B	B
RM243	1	7.97	A	B	B
RM1287	1	10.84	A	A	B
RM493	1	12.26	A	B	B
RM329	1	15.66	A	B	B
RM6711	1	16.44	A	B	B
RM10973	1	17.03	A	B	B
RM466	1	17.60	A	B	B
RM10998	1	18.02	A	B	B
RM7192	1	20.31	A	B	B
RM11110	1	20.67	A	B	B
RM5638	1	21.26	A	B	B
RM3341	1	22.21	A	B	B
RM11295	1	24.05	A	B	B
RM11408	1	26.18	A	B	B
RM443	1	28.34	A	B	B

RM3614	1	30.16	A	B	B
RM5954	1	30.32	A	B	B
RM11645	1	31.01	A	B	B
RM5914	1	31.84	A	B	B
RM543	1	32.78	A	B	B
RM212	1	33.05	A	B	B
RM3304	1	35.28	A	B	B
RM3825	1	36.80	A	A	B
RM1198	1	37.93	A	B	B
RM3602	1	39.34	A	B	B
RM3810	1	39.82	A	B	B
RM1387	1	40.53	A	A	B
RM6141	1	42.74	A	B	B
RM6321	1	43.25	A	B	B
RM6840	1	43.50	A	B	B
RM7451	2	0.65	A	B	B
RM5529	2	2.22	A	B	B
RM5553	2	4.67	A	B	B
RM6378	2	5.48	A	B	B
RM3227	2	7.47	A	A	B
RM3680	2	8.76	A	A	B
RM5791	2	10.75	A	B	B
RM3549	2	11.01	A	B	B
RM1234	2	11.34	A	B	B
RM1190	2	12.09	A	A	B
RM6374	2	15.18	A	A	B
RM5812	2	15.89	A	A	B
RM7426	2	16.67	A	A	B
RM3630	2	18.41	A	A	B
RM13294	2	18.64	A	B	B
RM13592	2	24.01	A	A	B
RM7271	2	25.82	A	B	B
RM6295	2	29.41	A	B	B
RM5993	2	29.70	A	B	B
RM3275	2	30.10	A	B	B
RM3263	2	32.66	A	A	B
RM1092	2	33.85	A	B	B
RM5412	3	0.44	A	B	B

RM3392	3	3.81	A	B	B
RM3417	3	5.63	A	B	B
RM5347	3	6.41	A	B	A
RM3716	3	6.82	A	B	B
RM6783	3	9.29	A	B	B
RM3803	3	10.64	A	B	B
RM6676	3	14.47	A	B	B
RM15240	3	17.73	A	B	B
RM411	3	21.39	A	B	B
RM3698	3	21.74	A	B	B
RM15450	3	23.09	A	B	B
RM5532	3	23.37	A	B	B
RM8209	3	28.84	A	B	B
RM1352	3	32.29	A	B	B
RM1230	3	32.70	A	B	B
In1	4	6.94	A	B	B
In2	4	6.97	A	B	B
RM16675	4	14.24	A	B	B
RM16792	4	18.00	A	B	B
RM3742	4	19.73	A	B	B
RM5979	4	20.58	A	B	B
RM3839	4	23.87	A	A	B
RM1388	4	25.00	A	B	B
RM241	4	25.81	A	B	B
RM6454	4	27.35	A	A	B
RM3474	4	29.68	A	B	B
RM3534	4	30.95	A	B	B
RM3687	4	31.26	A	B	B
RM3319	4	32.17	A	B	B
RM6238	4	32.79	A	B	B
RM7172	4	34.38	A	A	B
RM3683	5	7.16	A	B	B
RM6082	5	8.88	A	B	B
RM7409	5	9.22	A	B	B
RM3381	5	9.57	A	B	B
RM249	5	10.76	A	B	B
RM6645	5	15.00	A	B	B
RM18446	5	16.25	A	A	B

RM3351	5	20.68	A	B	B
RM3663	5	21.34	A	A	B
RM3295	5	22.25	A	A	B
RM19170	5	29.01	A	B	B
Sp1	6	1.63	A	A	B
RM6857	6	6.56	A	A	B
RM19720	6	8.06	A	B	B
RM6302	6	8.97	A	B	B
RM527	6	9.86	A	B	B
RM7213	6	10.60	A	A	B
RM20225	6	21.01	A	B	B
RM20589	6	28.01	A	B	B
RM3138	6	28.47	A	B	B
RM20646	6	29.04	A	B	B
RM5814	6	29.88	A	B	B
RM6652	7	0.58	A	B	B
RM21446	7	13.76	A	A	B
RM1135	7	16.93	A	A	B
RM432	7	18.96	A	B	B
RM3691	7	19.22	A	B	B
RM3404	7	20.11	A	B	B
RM21767	7	21.04	A	B	B
RM1365	7	23.79	A	B	B
RM1357	7	28.85	A	A	B
RM248	7	29.34	A	B	B
RM6356	8	1.56	A	B	B
RM3231	8	3.83	A	B	B
RM310	8	5.11	A	B	B
RM547	8	5.59	A	B	B
RM7267	8	10.18	A	B	B
RM22783	8	11.02	A	A	B
RM6471	8	12.38	A	A	B
RM404	8	15.31	A	A	B
RM7191	8	19.47	A	B	B
RM1109	8	20.35	A	A	B
RM23391	8	26.31	A	B	B
RM23654	9	0.15	A	A	B
RM23686	9	0.99	A	B	B

RM7481	9	6.59	A	B	B
RM24019	9	9.65	A	B	B
RM24097	9	11.02	A	B	B
RM24254	9	14.06	A	B	B
RM3700	9	15.43	A	B	B
RM3492	9	16.49	A	B	B
RM7175	9	16.87	A	B	B
RM6251	9	19.18	A	B	B
RM215	9	19.96	A	B	B
RM3808	9	20.55	A	B	B
RM5384	9	22.07	A	B	B
RM311	10	9.75	A	B	B
RM3229	10	16.43	A	B	B
RM5756	10	17.67	A	B	B
RM6474	10	18.37	A	B	B
RM6160	10	22.24	A	B	B
RM7453	11	0.93	A	B	B
RM6288	11	2.17	A	B	B
RM26259	11	6.00	A	A	B
RM7091	11	7.85	A	A	B
RM229	11	18.37	A	B	B
RM26896	11	19.93	A	B	B
RM26919	11	20.47	A	B	B
RM27108	11	24.05	A	A	B
RM6422	11	24.86	A	B	B
RM7221	11	26.79	A	A	B
RM27638	12	4.04	A	B	B
RM7619	12	4.83	A	B	B
RM3246	12	9.09	A	A	B
RM28313	12	20.02	A	A	B
BP26-Ind	12	22.87	A	B	B
RM5609	12	23.97	A	B	B
RM5479	12	24.38	A	A	B
RM235	12	26.11	A	B	B
