# Supplementary material for: Bph32, a novel gene encoding an unknown SCR domain-containing protein, confers resistance against the brown planthopper in rice
Source: Sci Rep. 2016 Nov 23;6:37645. doi: 10.1038/srep37645 (PMC5120289; doi:10.1038/srep37645)
Supplement: Supplementary Table S6 [file srep37645-s10.pdf]

Bph32, a novel gene encoding an unknown SCR domain-containing protein confers resistance against the brown planthopper in rice

Juansheng Ren[1]*, Fangyuan Gao[1]*, Xianting Wu[1]*, Xianjun Lu[1], Lihua Zeng[3], Jianqun Lv[1], Xiangwen Su[1], Hong Luo[2], and Guangjun Ren[1]**

[1]Crop Research Institute, Sichuan Academy of Agricultural Sciences, Chengdu, 610066, P.R. China

[2]Department of Genetics and Biochemistry, Clemson University,110 Biosystems Research Complex, Clemson, SC 29634-0318,USA

[3]Sichuan Normal University, Chengdu, 610066, P.R. China

*These authors contributed equally to the work.

**Corresponding author e-mail: guangjun61@sina.com

Table S6. List of the primers used in this paper.

Marker	Sequence(5´-3´)	Enzyme	Gene/fragment
WH1	TGAGGGAGTTGTAGTAGGAGTA<br>CGTCGTTGATGAAGTAAAGGT		LOC_Os06g03240（*Bph32*）
WB1	ATGGCAGCGATGATCGG<br>CTAATAGGTACAGACGTCGT		cDNA of *Bph32*
QB1	CACAAGCAAGAAATAAATGG<br>CACCAACTGCTTGCCG		Promoter of *Bph32b*
RP6	AGCGACCCTCAATGAACGGACTCT<br>ACGACTAAAACACGAATGACGGACC		Full-length *Bph32*
MB1	CCAAGCTTATGGCAGCGATGATCGG<br>CGAGCTCCTAATAGGTACAGACGTCGT	*Hind III*<br>*Sac I*	*Bph32*+PHB
MQ1	GCGTCGACCACAAGCAAGAAATAAATGG<br>GCTCTAGACACCAACTGCTTGCCG	*Sal I*<br>*Xba I*	Promoter of *Bph32*+P1300+PB1101
1108-Bph-ptb	GAATTCATGGCAGCGATGATCGGGACGCT<br>GGTACCATAGGTACAGACGTCGTTGATGGA	*EcoRI*<br>*KpnI*	pSAT6-mRFP-N1-*Bph32*
193F/486R	CGTGAGGGAGGACTACTGCTC<br>GATGATGCTGTAGAGCCTTGTG		*Bph32*
Sp1	CGAACAACTCCACGCTGT<br>GTGACGATGATGCTGTAGAG		*Bph32*
realt-Bph	TGGGTTCCGGTGGACCTGGG<br>GGACGTTGACCCTCGCCGTG		*Bph32*
Actin	gacggagcgtggttactcattc<br>gacctcagggcagcggaaa		*Actin*
qrt-Actin	CCACTATgTTCCCTggCATT<br>gTACTCAgCCTTggCAATCC		*Actin*
Bp3-In1	ATAGTGTATGAGGGTTGTTCTG<br>CATTCAACTTGGCTACACCT		*BPh3*
Bp3-In2	TATAGCCCTTGGAGAACG<br>GATCATGTCAGGAGAACCC		*Bph3*
Bp26-INd	TCCAAGGGAATGGTGTAGTAGCA<br>GGTCACTTCCAATTTGCGCAG		*BPH26*
